# Renal insufficiency among urban populations in Bangladesh: A decade of laboratory-based observations

**DOI:** 10.1371/journal.pone.0214568

**Published:** 2019-04-04

**Authors:** Sumon Kumar Das, Syeda Momena Afsana, Shahriar Bin Elahi, Mohammod Jobayer Chisti, Jui Das, Abdullah Al Mamun, Harold David McIntyre, Tahmeed Ahmed, Abu Syed Golam Faruque, Mohammed Abdus Salam

**Affiliations:** 1 icddr,b, Dhaka, Bangladesh; 2 Institute for Social Science Research, The University of Queensland, Brisbane, Qld, Australia; 3 Menzies—School of Health Research, Charles Darwin University, Darwin, Northern Territory, Australia; 4 School of Health and Rehabilitation Sciences, The University of Queensland, Brisbane, Qld, Australia; 5 Mater Clinical School, University of Queensland, Brisbane, Qld, Australia; 6 Mater Medical Research Institute, Raymond Tce, South Brisbane, Qld, Australia; Universidade Estadual Paulista Julio de Mesquita Filho, BRAZIL

## Abstract

**Objective:**

The objective of this study was to describe the age and sex-specific prevalence of renal insufficiency, and observe its trends over a decade at an urban Bangladesh setup.

**Method:**

This was a cross-sectional study, in which we observed the Estimated Glomerular Filtration Rate (eGFR) of 218,888 adults, aged ≥19 years, who had submitted their blood specimen to the Clinical Biochemistry Laboratory of the International Centre for Diarrhoeal Disease Research, Bangladesh (icddr,b) during the years 2006–2015. We applied CKD-EPI definition in estimating eGFR using their age-and sex-specific serum creatinine concentrations. Based on the eGFR, we classified the population into five stages of renal insufficiency (stage-1 to stage-5), at age intervals of five-years. Data were analysed using the Linear Regression and Multinomial Logistic Regression models.

**Results:**

Females constituted 43% (n = 94,931) of the study population; and 34% (n = 42,576) of the males and 31% (n = 29,830) of the females had their serum creatinine concentrations above the upper limit of the laboratory reference cut-off. The overall prevalence of stage-2 to stage-5 renal insufficiency were 24% (n = 52,126), 17% (n = 38,539), 8% (n = 16,504) and 6% (n = 12,665) respectively; the prevalence were 23% (n = 1,890), 19% (n = 1,579), 9% (n = 769) and 9% (n = 770) respectively in 2006, and 24% (n = 10,062), 17% (n = 6,903), 6% (n = 2,537) and 5% (n = 1,924) respectively in 2015. The prevalence was higher among the females. At least 2% of the adults, younger than <44 years, had stage-4 and stage-5 in 2015. The age-adjusted eGFR was significantly lower among the post-menopausal females (aged ≥46 y) compared to the same age group males (64.08±10.83 vs. 66.83±10.41 mL/min/1.73 m^2^; p<0.001). Compared to 2006, the number of individuals with renal insufficiency (stage 2 and above) had increased at least two times, irrespective of age, in 2015. A single year of increase in the age was significantly associated with 1.32 unit reductions in the eGFR; and the reductions were higher for females who also had higher odds of renal insufficiency stages-2 and beyond.

**Conclusion:**

This study observed high prevalence of stage-2 to stage-5 renal insufficiency in Bangladeshi populations, irrespective of age, and especially among the females.

## Background

The 2010 Global Burden of Disease Study ranked Chronic Kidney Disease (CKD) or renal insufficiency as the 18^th^ cause of global deaths, which was 27^th^ in 1990 [1]. This indicates a sharp rise in the prevalence of CKD over a two decade period making it the 3^rd^ highest cause of years of life lost, which can be translated as 82% increase in life years lost due to premature deaths attributable to CKD [1]. The burden is greater in the developing countries, particularly in Asia and sub-Saharan Africa, which also experienced a shift in the disease burden from infectious diseases to chronic illnesses, associated with reduced birth rate and increased life expectancy [2,3]. Taiwan, Japan, South Korea, Malaysia and India are among the top 20 countries experiencing the highest annual incidences of CKD [2]. Bangladesh is also in the list of rising annual prevalence of CKD or renal insufficiency.

The global review of six regions, including Bangladesh, reported an overall 14% prevalence of CKD [4]. A study in Dhaka, conducted among adults aged 30 years and older, reported a 26% prevalence of CKD [5], while other studies reported 13% prevalence among the urban Dhaka populations aged 15 years and older [6,7]. Another study reported CKD prevalence of at least 7% among the health service providers in Dhaka [8]. A community-based study estimated that 1/3^rd^ of the rural populations were at risk of CKD that largely remained undiagnosed [9]. However, due to the lack of comprehensive national health database, the changing trend of CKD remains unknown among Bangladeshi population. The reported variations in the prevalence of CKD among Bangladeshi populations may be due to cross-sectional study design using populations from different geographic locations within the Dhaka city, using populations of different ages, shorter study period, and modest sample size. The prevalence of CKD varies in different age groups, among males and females, and by socioeconomic conditions and rate of urbanization. Moreover, the prevalence of CKD is also influenced by health risk factors such as diabetes and hypertension [1,4].

The eGFR declines with age, due to the age related changes in the micro-anatomical structures of kidney and its functions [10,11], which explains increasing prevalence of CKD especially after 55 years of age. Reliable estimation of eGFR, as a measure of renal insufficiency, is influenced by ethnicity e.g. blood creatinine level that is related to muscle mass of individuals, might vary by ethnicity [12]. Currently, there is lack of reliable data to describe the age and sex specific creatinine levels and the prevalence of different stages of CKD among Bangladeshi populations, including those living in urban areas.

Taking the advantage of the large Clinical Laboratory database of the International Centre for Diarrhoeal Disease Research, Bangladesh (icddr,b), we conducted this study to understand the overall and the age-specific prevalence of different stages of CKD or renal insufficiency using estimated glomerular filtration rate (eGFR). We also studied the sex differentials and changing trends of different stages of renal insufficiency over a decade.

## Method

### Study design

This a cross-sectional study that extracted relevant data from the data archive of the Clinical Biochemistry Laboratory (CBL) of icddr,b.

### Study setting

Established in 1960, icddr,b is a premier health research institute in the global south. The Dhaka Hospital of icddr,b, established in 1962, currently provides care and treatment to nearly 150,000 patients with diarrhoeal illnesses, with or without complications and with or without associated health problems, each year. The research and the patient care activities of the icddr,b is supported by high-class CBL as well as other diagnostic and research laboratories. Being a research institution, icddr,b ensures high quality of tests performed by its various laboratories, which is also extensively used on a payment basis by the people living in the Dhaka city and its suburbs.

### Participants

For the purpose of our analyses, we extracted relevant data from the *Data Archive* of the Clinical Laboratory Services of icddr,b for the period of January 2006 through December 2015, and identified data of 243,187 adults, aged 19 years or older, relevant for the purpose of our study.

### Variables

The Database administrator provided anonymous data on the age, sex, date of laboratory assessment (day, month and year) and serum creatinine values of the individuals identified in the database, which included a small number of patients who were admitted to the Dhaka Hospital of icddr,b.

### Data measurement

Blood (either random or fasting) samples (5.0 mL) were collected for determining serum/ plasma creatinine concentrations. The serum creatinine was measured by kinetic colorimetric assay (Jaffe method, rate blanked and compensated) using the Hitachi 902 auto analyser from 2006 to 2009, and by an enzymatic method, using autoanalyzer Hitachi 902 and Olympus/ Beckman Coulter AU system from 2010.

### Study size

After excluding missing and extreme values, the analysable sample was 218,888.

### Quantitative variables

We estimated GFR using three different formulae: (i) Chronic Kidney Disease Epidemiology Collaboration (CKD-EPI); (ii) Modification of diet in renal disease (MDRD); and (iii) the Abbreviated MDRD formulae [13]. Due to the lack of information (e.g. on microalbuminuria) and inability to assess renal function, we were unable to follow the current definition of CKD [14]. Consequently, we followed the reference cut-off for the eGFR in defining five stages of renal insufficiency as follows: Stage 1 (representing normal kidney function with eGFR of ≥90 mL/min/1.73 m^2^; Stage-2 (mild renal insufficiency with eGFR of ≥ 60–89 mL/min.1.73 m^2^); Stage-3 (moderate renal insufficiency with eGFR of ≥ 30–59 mL/min/1.73 m^2^); Stage-4 (severe renal insufficiency with eGFR of ≥15–29 mL/min/1.73 m^2^) and Stage-5 (renal failure or end-stage renal disease [ESRD) with eGFR of <15 mL/min/1.73 m^2.^

We constituted age groups at 5 years interval, i.e. 19–24 years, 24–28 years, 29–33 years, 34–38 years, 39–43 years, 44–48 years, 49–53 years, 54–58 years, 59–63 years, 64–68 years, and 69 years and older.

### Ethics

Our research protocol for secondary analysis of de-identified data was approved by the Research Review Committee (RRC) and the Ethical Review Committee (ERC) of icddr,b.

### Statistical methods

We performed age and sex-specific analyses assuming progression of renal insufficiency as a function of age, and biological differences between males and females. The mean and standard deviations (SD), and median values of serum creatinine; and eGFR following three definitions, were determined for each of the age groups. The overall prevalence of high serum creatinine level was determined by considering individual’s serum creatinine value above the laboratory reference cut-off values (>106 mMol/L for males and >97 mMol/L for females). We also estimated the age group specific prevalence of five stages of renal insufficiency in 2006 and 2015, including their ratio. Several physiological changes occur in post-menopausal females, and we thus assumed 45 years as their menopausal age and used this cut-off in identifying possible differences in eGFR between pre-menopause (≤45 years) and post-menopause (>45 years) women. We compared the eGFR between the females and the males using the same age cut-off.

Finally, we performed unadjusted linear associations between eGFR and age, and estimated the Odds (OR) for developing different stages of renal insufficiency in different age groups by unadjusted multinomial logistic regression considering renal insufficiency stage-1 and people younger than 45 years as the reference groups. All analyses were performed separately for males and females, taking into consideration the three definitions of eGFR. We were able to extract concurrent blood glucose concentrations of 30,582 of our study population that enabled us perform subgroup analyses by estimating adjusted associations. All analyses were performed in STATA Version 13.0 (StataCorp, College Station, TX, USA).

## Results

**Fig 1:**
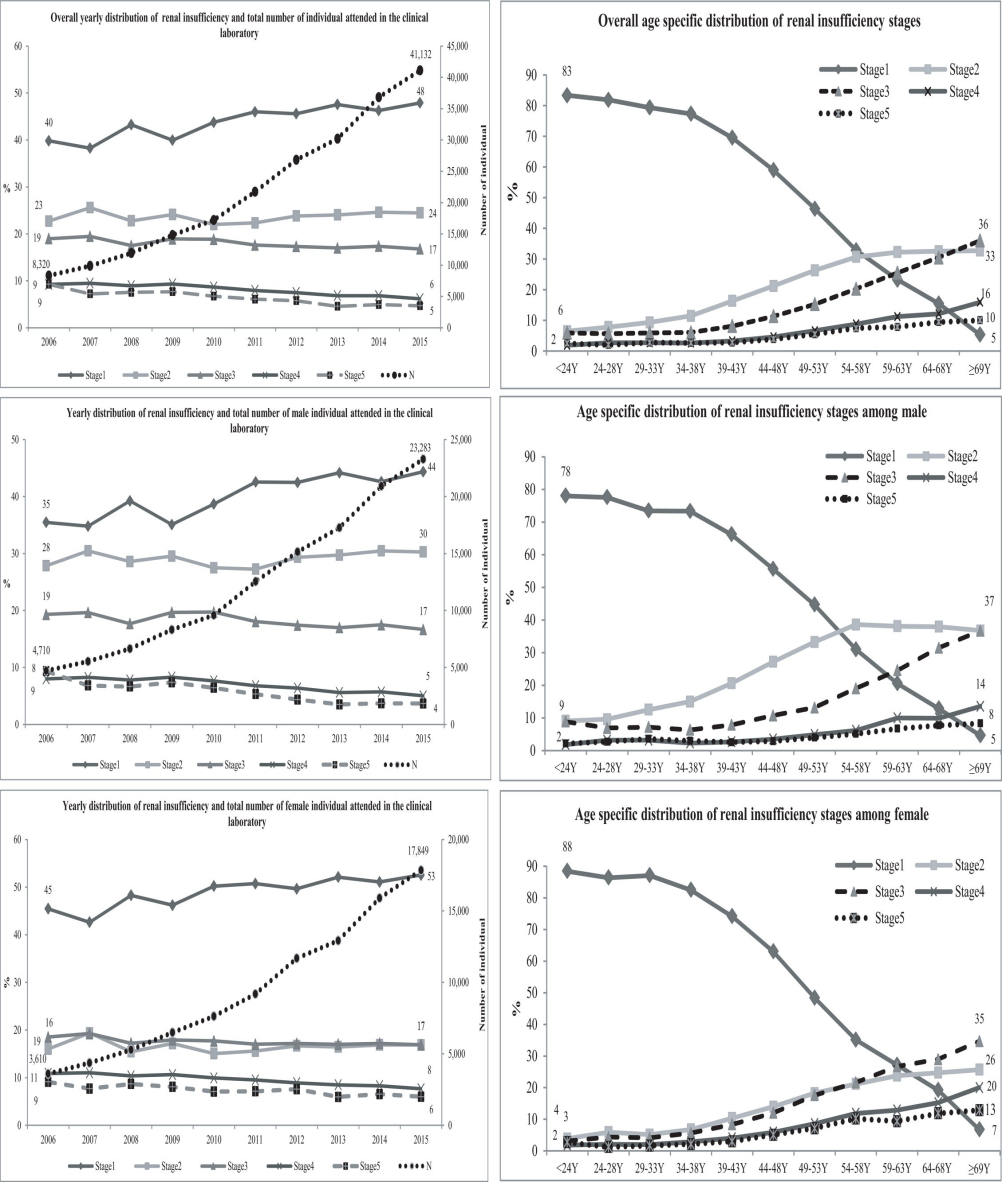
Overall and sex-specific distribution of renal insufficiency stages following CKD-EPI equation by year and by age groups.

Of the total analysable sample population, 94,931 (43%) were females. In 2006, a total of 8,320 individuals (43% females) attended the CBL for estimation of their serum creatinine. The number gradually increased over the years to reach 41,132 (4.93 times increased) in 2015 (Fig 1). The detailed age and year specific distributions of the study population are presented in S1 Table. The overall mean creatinine level in 2006 was 151.32 mMol/L (SD: 177.76; median: 89.7) and eGFR by CKD-EPI definition was 73.60 mL/min/1.73 m^2^ (SD: 40.51; median: 76.44). In 2015, these values were 117.95 mMol/L (SD 130.72; median: 82.5) and 82.32 mL/min.1.73 m^2^ (SD: 38.14; median: 85.12) respectively (detailed age-group specific yearly distributions of creatinine and eGFR following three definitions are provided in S2 Table). Around 34% (n = 42,576) of the males and 31% (n = 29,830) of the females had their serum creatinine levels higher than the upper limit of our laboratory reference cut-off. Following CKD-EPI definition, the overall prevalence of CKD stage-1 was 45% (n = 99,054), stage-2 was 24% (n = 52,126), stage-3 was 17% (n = 38,539), stage-4 was 8% (n = 16,504), and stage-5 was 6% (n = 12,665).

In 2006, the prevalence of renal insufficiency (according to CKD-EPI definition) stage-2 was 23% (n = 1,890), stage-3 was 19% (n = 1,579), stage-4 was 9% (n = 769) and stage-5 was 9% (n = 770); the corresponding population in 2015 were 24% (n = 10,062), 17% (n = 6,903), 6% (n = 2,537) and 5% (n = 1,924) respectively (Fig 1). These indicated a 5.3 times (10062/1890), 4.4 times (6903/1579), 3.3 time (2537/769), and 2.5 times (1924/770) increase in number of stage 2 to stage 5 renal insufficiency cases respectively over the period. The distributions of prevalence of renal insufficiency (CKD-EPI definition) among males and females are given in Fig 1. The corresponding stages of renal insufficiency based on eGFR as estimated by MDRD and Abbreviated MDRD equations are provided in S3 Table.

In the age-stratified analyses, the overall prevalence of renal insufficiency stage-1 reduced with increasing age (83% among individuals aged less than 24 years and 5% among 69 years and above), which commensurate with increased in the proportion with stages-2 through stage-5 of CKD (Fig 1). The prevalence of renal insufficiency stages-4 and 5 were higher among the females (Fig 1). The age stratified prevalence of renal insufficiency, as determined by MDRD and Abbreviated MDRD definitions are provided in S4 Table.

The detailed, age-stratified yearly prevalence of renal insufficiency, using all three definitions have been provided in Tables 1 and S5. Briefly, between 2006 and 2015, the prevalence of stage-2 renal insufficiency increased in all age groups, with the exceptions of those younger than 33 years. The prevalence of stage-3 renal insufficiency increased among people younger than 24 years and among those aged 59 years or older. Compared to 2006, the prevalence of stage-4 and stage-5 renal insufficiency reduced in all age group of males and females in 2015, although the absolute number of people with renal insufficiency increased over the period.

**Table 1:** Distribution of renal insufficiency stages in different age groups by CKD-EPI formula in 2006 and 2015.

			Stage-2		Stage-3		Stage-4		Stage-5		Overall*	Stage-2*	Stage-3*	Stage-4*	Stage-5*
Age group	Year	N	n	%	n	%	n	%	n	%					
*<24Y	2006	171	21	12.28	8	4.68	2	1.17	2	1.17	4.68	1.81	6.25	3.50	10.50
	2015	801	38	4.74	50	6.24	7	0.87	21	2.62					
24-28Y	2006	333	32	9.61	34	10.21	20	6.01	8	2.4	4.76	3.53	2.06	1.30	3.50
	2015	1,586	113	7.12	70	4.41	26	1.64	28	1.77					
29-33Y	2006	448	46	10.27	41	9.15	13	2.9	19	4.24	5.17	4.46	1.98	3.00	2.21
	2015	2,317	205	8.85	81	3.5	39	1.68	42	1.81					
34-38Y	2006	677	78	11.52	60	8.86	20	2.95	38	5.61	4.93	4.78	3.02	4.00	2.45
	2015	3,339	373	11.17	181	5.42	80	2.4	93	2.79					
39-43Y	2006	793	107	13.49	76	9.58	59	7.44	51	6.43	5.26	6.04	3.67	1.92	1.75
	2015	4,171	646	15.49	279	6.69	113	2.71	89	2.13					
44-48Y	2006	881	209	23.72	142	16.12	42	4.77	48	5.45	5.96	5.43	3.39	4.19	2.98
	2015	5,248	1,134	21.61	482	9.18	176	3.35	143	2.72					
49-53Y	2006	1,225	308	25.14	221	18.04	94	7.67	101	8.24	4.25	4.40	3.29	2.70	1.95
	2015	5,206	1,355	26.03	727	13.96	254	4.88	197	3.78					
54-58Y	2006	1,138	316	27.77	255	22.41	124	10.9	122	10.72	4.57	5.15	3.77	3.20	2.52
	2015	5,196	1,628	31.33	962	18.51	397	7.64	307	5.91					
59-63Y	2006	952	287	30.15	213	22.37	139	14.6	105	11.03	5.32	5.86	5.84	3.37	3.50
	2015	5,069	1,683	33.2	1,243	24.52	468	9.23	368	7.26					
64-68Y	2006	782	219	28.01	210	26.85	112	14.32	127	16.24	4.71	5.87	5.45	3.45	1.98
	2015	3,680	1,286	34.95	1,144	31.09	386	10.49	252	6.85					
≥69Y	2006	920	267	29.02	319	34.67	144	15.65	149	16.2	4.91	6.00	5.28	4.10	2.58
	2015	4,519	1,601	35.43	1,684	37.26	591	13.08	384	8.5					

*ratio of overall number of individual assessed serum creatinine and number of CKD stage 2 to stage-5 between 2015 and 2006

According to the CKD-EPI definition, we noted an inverse relation between age and mean eGFR (Table 2). There were 1.32 unit (mL/min/1.73 m^2^) reductions in the eGFR by single year increase in age, and the reductions were higher among the females compared to males (1.56 vs. 1.15 mL/min/1.73 m^2^) and they did not significantly change after adjusting for blood glucose (Table 2). In the multinomial logistic regression analyses, significantly higher ORs were observed for all stages of renal insufficiency (stage-2 to stage-5) in all age group (Table 3), and the ORs increased after adjusting for blood glucose (S8 Table). The proportion of individuals with renal insufficiency, as defined by estimation of eGFR by MDRD and Abbreviated MDRD definitions, are presented in S6 and S7 Tables.

**Table 2:** Overall and sex-specific association of eGFR following CKD-EPI formula.

	Coef.	95% CI	
		LL	UL
^*^Overall (N = 218888)	-1.32	-1.33	-1.31
Male (N = 123957)	-1.15	-1.16	-1.14
Female (N = 94931)	-1.56	-1.57	-1.55
***Adjusted for blood glucose***			
^#^Overall (N = 30582)	-1.35	-1.37	-1.33
Male (N = 18165)	-1.20	-1.23	-1.18
Female (N = 12417)	-1.58	-1.62	-1.54

^*^adjusted for age, sex and years;

^#^adjusted for age, sex, years and blood glucose.

Sex was removed in sex specific analyses

**Table 3:** Overall and sex-specific risk of renal insufficiency following CKD-EPI formula.

	Overall (N = 218888)			Male (N = 123957)			Female (N = 94931)		
	OR	95% CI		OR	95% CI		OR	95% CI	
Stage-2		LL	UL		LL	UL		LL	UL
<45Y	Ref.	Ref.	Ref.	Ref.	Ref.	Ref.	Ref.	Ref.	Ref.
45-48Y	2.34	2.25	2.44	2.25	2.14	2.36	2.49	2.32	2.67
49-53Y	3.64	3.50	3.77	3.31	3.17	3.47	4.10	3.85	4.37
54-58Y	6.00	5.79	6.23	5.56	5.30	5.82	6.58	6.18	7.01
59-63Y	8.68	8.34	9.04	8.32	7.91	8.76	9.59	8.96	10.26
64-68Y	13.18	12.54	13.86	13.24	12.39	14.15	14.10	13.01	15.28
≥69Y	37.01	34.73	39.45	35.96	33.08	39.10	41.91	37.88	46.36
Stage-3									
<45Y	Ref.	Ref.	Ref.	Ref.	Ref.	Ref.	Ref.	Ref.	Ref.
45-48Y	2.27	2.15	2.38	1.94	1.81	2.09	2.73	2.53	2.94
49-53Y	3.71	3.54	3.88	2.84	2.67	3.03	4.95	4.62	5.29
54-58Y	6.98	6.67	7.29	5.93	5.58	6.29	8.42	7.88	9.00
59-63Y	12.42	11.86	13.00	11.68	10.99	12.41	13.55	12.63	14.53
64-68Y	22.35	21.18	23.59	23.99	22.30	25.81	20.73	19.11	22.48
≥69Y	75.56	70.73	80.72	78.31	71.73	85.50	71.14	64.32	78.69
Stage-4									
<45Y	Ref.	Ref.	Ref.	Ref.	Ref.	Ref.	Ref.	Ref.	Ref.
45-48Y	2.26	2.09	2.44	1.76	1.57	1.97	2.84	2.56	3.15
49-53Y	3.82	3.57	4.08	2.87	2.60	3.16	4.97	4.53	5.45
54-58Y	7.20	6.76	7.66	5.25	4.79	5.75	9.52	8.72	10.39
59-63Y	13.29	12.48	14.16	13.05	11.97	14.23	13.58	12.39	14.89
64-68Y	21.63	20.14	23.23	20.88	18.89	23.08	22.55	20.37	24.96
≥69Y	82.91	76.68	89.63	79.84	71.82	88.75	85.06	75.79	95.46
Stage-5									
<45Y	Ref.	Ref.	Ref.	Ref.	Ref.	Ref.	Ref.	Ref.	Ref.
45-48Y	1.98	1.83	2.15	1.39	1.23	1.57	2.89	2.58	3.24
49-53Y	3.36	3.13	3.61	2.23	2.01	2.47	5.13	4.64	5.67
54-58Y	6.52	6.10	6.96	4.13	3.76	4.55	10.15	9.23	11.17
59-63Y	9.95	9.30	10.66	8.47	7.72	9.29	12.21	11.02	13.53
64-68Y	18.00	16.69	19.41	15.62	14.07	17.35	21.69	19.41	24.23
≥69Y	55.92	51.48	60.74	47.37	42.40	52.91	67.90	59.93	76.92

NB: Stage -1 considered as reference group; adjusted for sex and years; sex was removed in sex specific analyses

OR: Odds ratio; CI: Confidence interval; LL: Lower limit; UL: Upper limit; Y: Years

**Fig 2:**
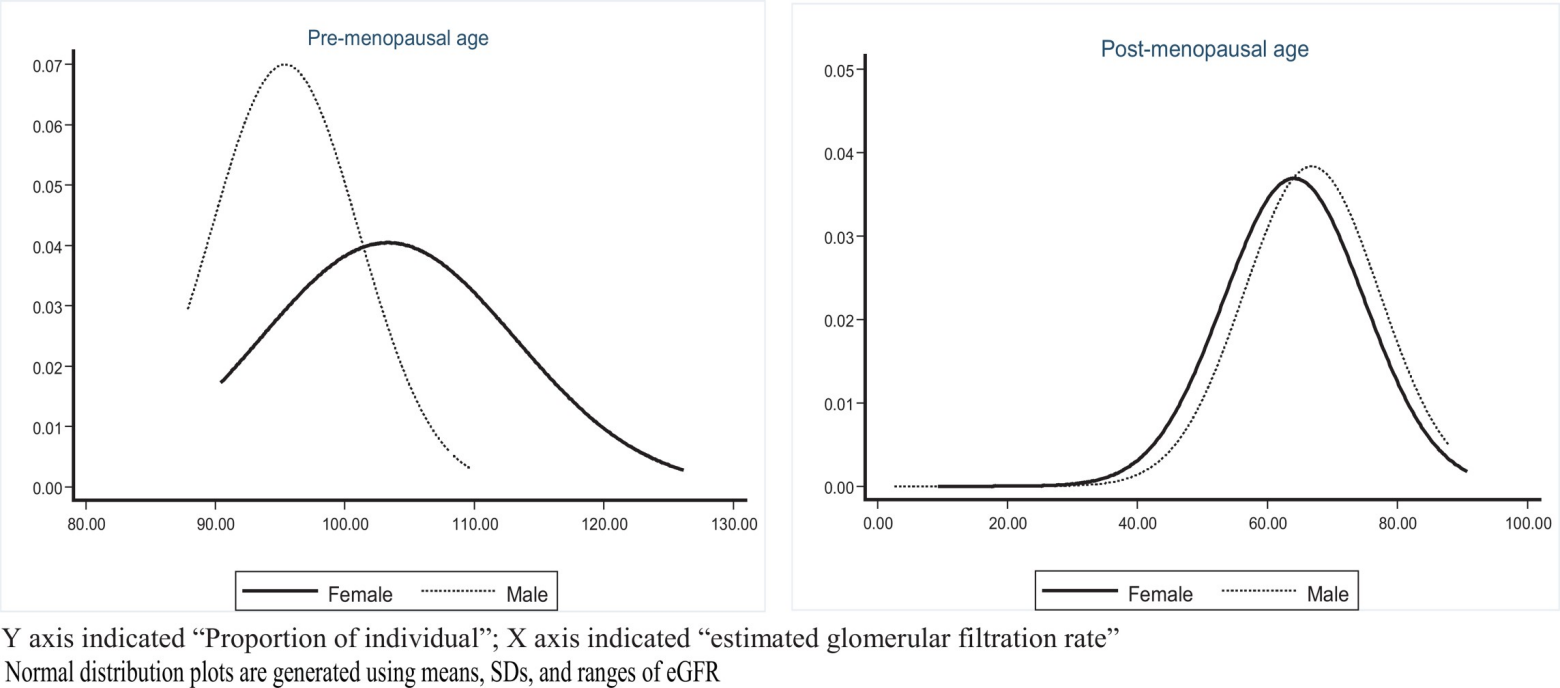
Distribution of eGFR at menopausal age among females and males at same age.

Fig 2 describes the changes in the mean eGFR among the females before and after their menopausal age, and males using the same age cut-off. Pre-menopausal women had significantly higher mean eGFR than the males (103.32±9.86 vs. 95.38±5.70; p<0.001); however, the post-menopausal women had lower mean eGFR than the same age group males (64.08±10.83 vs. 66.83±10.41; p<0.001).

## Discussion

The kidneys are vital organ that is primarily responsible for excretion of waste metabolites through urine, and maintenance of water and electrolyte balances. Impairment of kidney function most often remains undiagnosed because it is not routinely assessed, and delayed disease manifestation as only 10% of the total nephrons may maintain homeostasis. Thus, the diagnosis of CKD at its early stage is often incidental, and usually diagnosed while assessing optimal renal function before surgery or prescribing new medications, or during assessment of other co-morbidities.

In our study, around 55% of the individuals had laboratory evidence of renal insufficiency (stage-2 and above) following CKD-EPI equation for estimation of GFR; the prevalences were greater using MDRD (58%) and Abbreviated MDRD (64%) equations were applied.

Generally, renal insufficiency or CKD is a disease among elderly as there is an average yearly loss of 0.4% of the total glomeruli after the age of 20 years [15]. After 40 years of age the GFR reduces at a rate of 0.94 mL/min/1.73 m^2^. This supports our observed inverse linear association of eGFR with increasing age and higher prevalence of renal insufficiency among the elderly (aged 60 years and older). However, a proportion of our middle-age people had evidence of stage 4–5 renal insufficiency, which is also not uncommon among individuals younger than 40 years. We noted an overall high prevalence of stage-2 and stage-3 renal insufficiency with trends of increase with increasing age; however, the overall yearly prevalence did not significantly increase. In fact, we observed a reducing trend in the prevalence of renal insufficiency stages 2 through 5. The number of individuals who had an assessment of their serum creatinine increased by 5 folds in 2015 compared to 2006 (detailed described under Tables 1 and S5) and the absolute number of renal insufficiency cases had doubled or tripled in every age group over the study period. This might indicate that determination of serum creatinine is done more often even in people with no insufficiency reducing the prevalence, or re-testing of the same individual is more frequent. However, we were unable to identify the real cause from our data set due to the lack of required information, and true prevalence can only be estimated based on a carefully conducted national survey.

In 2015, serum creatinine was estimated for a total of 41,132 individuals and 1,924 (4.7%) had stage-5 renal insufficiency (renal failure or ESRD) among which 194 (10%) were younger than 39 years. Among 2,537 (6.2% of the total) people with stage-4 renal insufficiency, 152 (6%); and among 6,903 (16.8% of total) with stage-3, 382 (6%) were younger than 39 years. Most of our study people self-referred to CBL for performing various laboratory tests including estimating serum creatinine. Thus, the proportion of these young people with moderate to mild renal insufficiency (stage-3 and stage-2) were unexpectedly high and likely they were unaware of their conditions. We also noted that 1/3^rd^ of our total study population (both sexes) had serum creatinine concentrations (easier to understand by lay people) above our laboratory reference values. These observations suggest the possibility of additional future risk of cardiovascular disease and deaths among this population, as reported earlier among persons with renal insufficiency or CKD, especially those with microalbuminuria [16]. However, we do not have information on microalbuminuria among our study population and thus unable to estimate their risk.

Diabetes is the major cause of renal insufficiency or CKD [1]. We performed separate analyses for a sub-group of the study population who had their blood glucose estimated, and observed higher risk of renal insufficiency among them (Tables 2 and S8). Hypertension, obesity and high cholesterol are among the other major factors for progression of CKD [1,4]. We could not study the association of these factors in our population, due to the lack of such information; however, Bangladesh is now experiencing increasing burden of all these chronic morbidities; according to Bangladesh Health and Demographic Survey (BDHS) 2011, 32% women and 19% men were hypertensive, 11% (both sexes) had diabetes, and 17% of men and 8% of women were obese/overweight [16]. Another study reported a higher prevalence of hyperlipidaemia, irrespective of age and sex, among the urban population in Bangladesh [17]. The higher prevalence of all the chronic co-morbid risk factors with changing sedentary lifestyle and dietary behaviour and overcrowded residence [18,19] make the urban populations at future risk of renal insufficiency. A recent study has reported higher prevalence of the disease among the poorest sectors of population [1], which poses an additional risk to people living in Bangladesh where a large proportion living in the slums are poor. However, our study population are not likely to represent urban poor. The use of herbal medication (many uses toxic heavy metals) and environmental toxins [1] are among other potential offenders, and their use is not uncommon in Bangladesh. Due to the lack of information of socio-demography (including family history) and life-style factors in our CBL database, we could not explore such risks. Rapid urbanization and increasing elderly population are growing concern [20,21]. Additionally, recent food adulteration by using toxic substance, dyes, heavy metals, and irrational use of drugs might be the other possible reasons mostly attributable to renal damage [22].

Bangladesh National Survey 2011 also disclosed higher prevalence of all chronic co-morbid predictors (such as hypertension, diabetes and overweight and obesity) of renal insufficiency among the females [16], which corroborates with our findings of higher prevalence of renal insufficiency stages-2 and beyond among our female population. Hormonal changes, especial during post-menopausal period, could be a factor to explain higher prevalence of renal insufficiency among our study females. We also observed significantly lower eGFR in post-menopausal females compared to males of the same age group.

## Strengths and limitations

The large sample size (population aged 19 years or older) and availability of quality laboratory data over a longer period are the main strengths of our study. There is limited information on serum creatinine levels in young adults (to study eGFR status) and prevalence of renal insufficiency stages—our study had been blessed with availability of good number of such people. We extracted data from the CBL database where both apparently healthy people and individuals with suspected/ known renal insufficiency or other conditions, either self-referred or referred by family physicians, and assessed their serum creatinine level. The CBL (similar to other laboratories in Bangladesh) do not ask for diagnosis or clinical conditions from self-referred individuals; and more importantly, the referring physicians generally do not provide clinical diagnosis or even pertinent clinical findings in their referral notes. Moreover, even when such information is available, our CBL do not routinely collect such information in its database. Thus, we were unable to get useful information on potential factors that are significantly associated with renal insufficiency, such as life-style (e.g. smoking, diet, alcohol use, calorie consumption, physical activity at work and/or leisure) and socio-demographic (except age and sex) factors. Additionally, we could not collect information on morbidities that are associated with an acute rise of serum creatinine level for our evaluation. Since persons referred themselves or by their physicians it can assumed that they were unwell and that the underlying illnesses could have contributed to their impaired kidney function. The majority of study individuals were urban residents coming from different locations within the Dhaka city (only a few from peri-urban areas), which addresses a limitation of earlier studies. Poor people were likely underrepresented in our study and thus our findings are biased towards well-to-do people who are able to afford testing on payment- another limitation of our study. It is possible that we included multiple results of the same individual collected at different time points in our study period—we could not identify such people due to the lack of ID linkages in the laboratory registration system. Consequently, our estimates of various stages of renal insufficiency could be an over-estimate as well underestimate. Lack of information, especially on microalbuminuria, a less invasive and cost effective test, is another limitation for defining CKD [14]. Additionally, single assessment of serum creatinine clearly does not meet the requirements for the definition of CKD. It is essential to have abnormal serum creatinine on 2 occasions at least three-months apart. High-quality of laboratory assays is our strength; however, using two different methods for estimation of creatinine (compensated Jaffe method till 2009 and enzymatic method since 2010) might have impacted the results although their magnitude remains unknown while both are valid methods for measuring serum creatinine. Due to the lack of validation study about the best equation for estimating GFR among our population (though CKD-EPI is the most updated equation which considers age, sex and ethnicity), we use three equations. The findings on renal insufficiency across three equations were undistinguishable. Finally, it should also be emphasised that the majority of individuals with renal insufficiency stage 2 and stage 3 may not progress to end-stage kidney failure.

## Conclusion

The results of our study suggest that in a country like Bangladesh where the general population is still deprived of minimal primary care, likely there is a huge burden of renal insufficiency including renal failure or ESRD. Individuals with advanced renal insufficiency need highly expensive dialysis and kidney transplantation services, which are still out of the reach of the most of the Bangladesh population. Due to the lack of any national chronic disease monitoring system and awareness programmes, most people with renal insufficiency/ CKD are not even aware of their conditions and consequently unable to take measures for minimizing further damage to their kidneys and avoiding catastrophes. We observed that young adults are also at risk of renal function impairment and development of renal insufficiency, which calls for greater efforts for early diagnosis of diabetes and hypertension, and control of obesity and hypercholesterolemia–conditions that are known contributors to renal insufficiency. Finally, our finding of higher risk of renal insufficiency among females, especially those in post-menopausal age, points to a need for additional awareness on early life modification for prevention and control of renal insufficiency or CKD.

## Supporting information

S1 Table Yearly age-group specified distribution of individual attended in the clinical laboratory. (DOCX)

S2 Table Yearly age-specific distribution of serum creatinine and estimated glomerular filtration rate following three definitions. (DOCX)

S3 Table Overall and sex-specific yearly distribution of renal insufficiency stages following MDRD and abbreviated MDRD equations. (DOCX)

S4 Table Age-stratified prevalence of renal insufficiency stages following MDRD and abbreviated MDRD equations. (DOCX)

S5 Table Distribution of renal insufficiency stages in different age groups by MDRD and abbreviated MDRD equations in 2006 and 2015. (DOCX)

S6 Table Overall and sex-specific association of eGFR following MDRD and Abbreviated MDRD equations. (DOCX)

S7 Table Overall and sex-specific risk of renal insufficiency following MDRD and abbreviated MDRD equations. (DOCX)

S8 Table Overall and sex-specific blood glucose adjusted risk of renal insufficiency following CKD-EPI equations. (DOCX)

## Abbreviations

CBL: Clinical Biochemistry Laboratory
CKD: Chronic kidney disease
CKD-EPI: Chronic Kidney Disease Epidemiology Collaboration
eGFR: Estimated glomerular filtration rate
ESRD: End stage of Kidney Disease
icddr,b: International Centre for Diarrhoeal Disease research, Bangladesh
MDRD: Modification of diet in renal disease
OR: Odds ratio
