# Supplementary material for: Renal insufficiency among urban populations in Bangladesh: A decade of laboratory-based observations
Source: PLoS One. 2019 Apr 4;14(4):e0214568. doi: 10.1371/journal.pone.0214568 (PMC6448896; doi:10.1371/journal.pone.0214568)
Supplement: S1 Table — (DOCX) [file pone.0214568.s001.docx]

**S1 Table:** Yearly age-group specified distribution of individual attended in the clinical laboratory

| **Year** | **<24Y** | **24-28Y** | **29-33Y** | **34-38Y** | **39-43Y** | **44-48Y** | **49-53Y** | **54-58Y** | **59-63Y** | **64-68Y** | **≥69Y** | **Total** |
| --- | --- | --- | --- | --- | --- | --- | --- | --- | --- | --- | --- | --- |
| 2006 | 171 | 333 | 448 | 677 | 793 | 881 | 1,225 | 1,138 | 952 | 782 | 920 | 8,320 |
| 2007 | 241 | 465 | 522 | 760 | 892 | 1,157 | 1,342 | 1,400 | 1,144 | 872 | 1,101 | 9,896 |
| 2008 | 281 | 522 | 638 | 1,005 | 1,184 | 1,377 | 1,544 | 1,689 | 1,366 | 1,050 | 1,276 | 11,932 |
| 2009 | 441 | 749 | 872 | 1,181 | 1,432 | 1,639 | 1,975 | 1,993 | 1,719 | 1,194 | 1,606 | 14,801 |
| 2010 | 448 | 746 | 1,007 | 1,358 | 1,750 | 1,957 | 2,240 | 2,393 | 2,094 | 1,387 | 1,834 | 17,214 |
| 2011 | 565 | 947 | 1,287 | 1,659 | 2,399 | 2,565 | 2,763 | 3,030 | 2,495 | 1,693 | 2,336 | 21,739 |
| 2012 | 569 | 1,169 | 1,573 | 2,134 | 2,685 | 3,353 | 3,443 | 3,585 | 3,237 | 2,279 | 2,803 | 26,830 |
| 2013 | 652 | 1,279 | 1,840 | 2,370 | 3,220 | 3,619 | 3,901 | 3,902 | 3,682 | 2,453 | 3,285 | 30,203 |
| 2014 | 705 | 1,356 | 2,121 | 2,898 | 3,731 | 4,621 | 4,681 | 4,862 | 4,547 | 3,233 | 4,066 | 36,821 |
| 2015 | 801 | 1,586 | 2,317 | 3,339 | 4,171 | 5,248 | 5,206 | 5,196 | 5,069 | 3,680 | 4,519 | 41,132 |
| **Total** | 4,874 | 9,152 | 12,625 | 17,381 | 22,257 | 26,417 | 28,320 | 29,188 | 26,305 | 18,623 | 23,746 | 218,888 |
