# Supplementary material for: Renal insufficiency among urban populations in Bangladesh: A decade of laboratory-based observations
Source: PLoS One. 2019 Apr 4;14(4):e0214568. doi: 10.1371/journal.pone.0214568 (PMC6448896; doi:10.1371/journal.pone.0214568)
Supplement: S2 Table — (DOCX) [file pone.0214568.s002.docx]

**S2 Table:** Yearly age-specific distribution of serum creatinine and estimated glomerular filtration rate following three equations

| S. Creatinine | 19-24Y (4,874) | | 24-28Y (9,152) | | 29-33Y (12,625) | | 34-38Y (17,381) | | 39-43Y (22,257) | | 44-48Y (26,417) | | 49-53Y (28,320) | | 54-58Y (29,188) | | 59-63Y (26,305) | | 64-68Y (18,623) | | ≥69Y (23,746) | |  |
| --- | --- | --- | --- | --- | --- | --- | --- | --- | --- | --- | --- | --- | --- | --- | --- | --- | --- | --- | --- | --- | --- | --- | --- |
|  | Mean | sd | Mean | sd | Mean | sd | Mean | sd | Mean | sd | Mean | sd | Mean | sd | Mean | sd | Mean | sd | Mean | sd | Mean | sd | |
| 2006 | 87.56 | 116.22 | 108.02 | 138.91 | 118.28 | 179.65 | 116.63 | 162.09 | 137.97 | 189.46 | 127.70 | 174.50 | 137.16 | 137.63 | 166.70 | 189.19 | 175.91 | 197.33 | 197.15 | 199.12 | 190.02 | 170.58 | |
| 2007 | 88.76 | 92.82 | 94.47 | 95.71 | 116.49 | 138.31 | 100.93 | 133.38 | 118.47 | 149.66 | 120.77 | 135.48 | 128.85 | 114.77 | 149.26 | 152.21 | 170.27 | 170.28 | 180.38 | 171.04 | 179.58 | 157.19 | |
| 2008 | 104.90 | 195.13 | 97.69 | 106.24 | 108.00 | 139.77 | 100.41 | 143.68 | 104.57 | 135.46 | 132.08 | 176.38 | 139.39 | 152.93 | 151.11 | 159.45 | 149.09 | 134.88 | 176.66 | 172.20 | 175.74 | 154.78 | |
| 2009 | 108.10 | 129.92 | 105.85 | 143.77 | 112.07 | 154.75 | 103.99 | 126.71 | 108.48 | 123.95 | 127.17 | 159.60 | 144.07 | 152.73 | 161.67 | 177.83 | 160.03 | 145.13 | 179.48 | 188.05 | 180.98 | 162.95 | |
| 2010 | 97.92 | 135.91 | 87.42 | 100.98 | 103.60 | 139.44 | 103.72 | 123.14 | 105.45 | 114.73 | 119.69 | 148.59 | 135.81 | 141.47 | 152.14 | 176.78 | 159.64 | 157.46 | 155.84 | 158.49 | 167.21 | 141.52 | |
| 2011 | 95.28 | 131.29 | 97.61 | 116.47 | 110.05 | 173.13 | 109.69 | 156.50 | 99.98 | 117.33 | 112.69 | 127.73 | 128.97 | 155.70 | 143.67 | 152.36 | 146.31 | 150.60 | 168.41 | 175.61 | 163.55 | 132.88 | |
| 2012 | 84.34 | 101.17 | 91.23 | 101.78 | 92.13 | 108.56 | 91.85 | 111.75 | 103.53 | 122.36 | 114.83 | 134.41 | 124.87 | 145.76 | 142.70 | 152.61 | 146.89 | 148.24 | 159.39 | 153.87 | 158.26 | 130.58 | |
| 2013 | 92.95 | 132.57 | 90.76 | 135.61 | 90.92 | 101.13 | 90.11 | 110.11 | 98.71 | 118.07 | 104.84 | 114.54 | 112.59 | 110.18 | 128.15 | 132.38 | 142.30 | 143.62 | 143.17 | 133.37 | 148.25 | 122.60 | |
| 2014 | 89.43 | 122.44 | 88.52 | 111.35 | 98.77 | 119.36 | 93.84 | 118.94 | 94.35 | 95.09 | 107.20 | 125.62 | 117.12 | 134.71 | 132.83 | 139.00 | 141.82 | 138.62 | 147.37 | 135.96 | 151.49 | 133.38 | |
| 2015 | 95.59 | 153.10 | 88.11 | 122.52 | 88.10 | 113.16 | 99.39 | 134.01 | 94.76 | 103.98 | 102.68 | 122.94 | 111.42 | 121.53 | 128.57 | 138.38 | 138.99 | 137.18 | 145.14 | 145.18 | 150.10 | 131.52 | |
| eGFR (MDRD) | |  |  |  |  |  |  |  |  |  |  |  |  |  |  |  |  |  |  |  |  |  | |
| 2006 | 118.03 | 39.83 | 104.49 | 45.45 | 101.81 | 43.30 | 96.09 | 40.55 | 86.62 | 40.33 | 79.90 | 35.67 | 72.20 | 35.01 | 64.71 | 35.60 | 60.13 | 34.50 | 53.54 | 32.54 | 50.50 | 30.26 | |
| 2007 | 119.82 | 46.06 | 104.87 | 37.31 | 94.78 | 40.09 | 97.00 | 33.55 | 87.22 | 34.66 | 79.46 | 35.08 | 70.40 | 32.31 | 64.13 | 31.69 | 58.72 | 32.17 | 52.89 | 30.97 | 50.01 | 28.28 | |
| 2008 | 119.50 | 47.70 | 109.57 | 43.41 | 103.06 | 40.55 | 99.27 | 33.10 | 93.34 | 36.29 | 81.73 | 36.28 | 73.50 | 36.74 | 66.08 | 33.85 | 61.73 | 32.57 | 55.19 | 31.80 | 53.20 | 31.49 | |
| 2009 | 108.37 | 46.69 | 107.31 | 43.34 | 101.41 | 43.12 | 96.18 | 36.89 | 89.82 | 36.08 | 81.05 | 36.40 | 70.12 | 36.00 | 64.48 | 34.90 | 57.68 | 31.53 | 55.49 | 31.87 | 50.99 | 31.03 | |
| 2010 | 120.01 | 46.61 | 113.52 | 40.07 | 103.45 | 39.85 | 98.92 | 39.50 | 90.92 | 34.92 | 84.20 | 36.77 | 72.98 | 36.34 | 67.38 | 34.31 | 60.86 | 32.80 | 58.50 | 31.36 | 53.14 | 30.34 | |
| 2011 | 124.97 | 51.87 | 111.72 | 43.90 | 106.12 | 43.69 | 100.37 | 40.45 | 96.79 | 37.08 | 87.66 | 38.61 | 78.46 | 36.24 | 70.16 | 36.16 | 64.68 | 32.01 | 58.10 | 32.34 | 53.38 | 30.08 | |
| 2012 | 122.98 | 42.23 | 112.36 | 41.58 | 107.93 | 37.75 | 102.97 | 35.31 | 93.62 | 34.83 | 86.04 | 36.18 | 78.90 | 35.18 | 68.53 | 34.29 | 64.05 | 31.64 | 58.43 | 31.39 | 54.03 | 29.11 | |
| 2013 | 123.49 | 40.78 | 115.96 | 40.27 | 108.44 | 37.81 | 103.96 | 32.83 | 95.58 | 33.47 | 88.63 | 34.57 | 80.35 | 33.77 | 72.06 | 33.77 | 65.15 | 32.13 | 61.76 | 30.71 | 56.51 | 28.86 | |
| 2014 | 120.58 | 39.91 | 113.94 | 36.74 | 105.79 | 37.97 | 102.08 | 32.91 | 95.36 | 32.88 | 88.53 | 34.38 | 80.98 | 34.10 | 70.60 | 33.26 | 64.40 | 31.21 | 60.28 | 30.09 | 56.36 | 28.57 | |
| 2015 | 121.69 | 43.38 | 115.65 | 38.92 | 109.85 | 34.71 | 101.61 | 35.66 | 96.94 | 33.47 | 90.45 | 33.94 | 82.74 | 34.29 | 72.73 | 33.45 | 66.52 | 32.88 | 62.01 | 30.26 | 57.24 | 29.93 | |
| eGFR (Abb-MDRD) | |  |  |  |  |  |  |  |  |  |  |  |  |  |  |  |  |  |  |  |  |  | |
| 2006 | 111.27 | 37.56 | 98.50 | 42.85 | 95.97 | 40.83 | 90.58 | 38.23 | 81.65 | 38.03 | 75.32 | 33.63 | 68.06 | 33.01 | 61.00 | 33.56 | 56.67 | 32.52 | 50.47 | 30.67 | 47.60 | 28.52 | |
| 2007 | 112.96 | 43.42 | 98.86 | 35.18 | 89.35 | 37.80 | 91.43 | 31.64 | 82.22 | 32.68 | 74.90 | 33.07 | 66.35 | 30.46 | 60.45 | 29.87 | 55.35 | 30.33 | 49.85 | 29.19 | 47.13 | 26.65 | |
| 2008 | 112.67 | 44.99 | 103.30 | 40.95 | 97.15 | 38.24 | 93.57 | 31.21 | 87.98 | 34.21 | 77.04 | 34.20 | 69.28 | 34.64 | 62.28 | 31.91 | 58.18 | 30.71 | 52.02 | 29.97 | 50.14 | 29.68 | |
| 2009 | 102.17 | 44.04 | 101.16 | 40.88 | 95.60 | 40.66 | 90.66 | 34.78 | 84.66 | 34.01 | 76.40 | 34.32 | 66.10 | 33.94 | 60.78 | 32.90 | 54.37 | 29.72 | 52.31 | 30.04 | 48.06 | 29.24 | |
| 2010 | 113.14 | 43.95 | 107.03 | 37.80 | 97.52 | 37.58 | 93.25 | 37.24 | 85.71 | 32.92 | 79.37 | 34.67 | 68.80 | 34.26 | 63.51 | 32.35 | 57.36 | 30.91 | 55.14 | 29.56 | 50.08 | 28.59 | |
| 2011 | 117.81 | 48.89 | 105.31 | 41.39 | 100.04 | 41.20 | 94.61 | 38.14 | 91.23 | 34.96 | 82.63 | 36.40 | 73.96 | 34.16 | 66.14 | 34.09 | 60.96 | 30.17 | 54.76 | 30.48 | 50.30 | 28.35 | |
| 2012 | 115.94 | 39.83 | 105.92 | 39.21 | 101.74 | 35.60 | 97.06 | 33.30 | 88.24 | 32.83 | 81.10 | 34.11 | 74.38 | 33.16 | 64.59 | 32.32 | 60.37 | 29.82 | 55.07 | 29.58 | 50.93 | 27.44 | |
| 2013 | 116.41 | 38.46 | 109.32 | 37.98 | 102.22 | 35.66 | 98.00 | 30.96 | 90.09 | 31.55 | 83.54 | 32.59 | 75.73 | 31.83 | 67.92 | 31.83 | 61.41 | 30.28 | 58.21 | 28.94 | 53.26 | 27.20 | |
| 2014 | 113.68 | 37.64 | 107.42 | 34.65 | 99.72 | 35.80 | 96.22 | 31.03 | 89.88 | 31.00 | 83.45 | 32.42 | 76.34 | 32.15 | 66.55 | 31.35 | 60.70 | 29.42 | 56.82 | 28.36 | 53.12 | 26.92 | |
| 2015 | 114.71 | 40.90 | 109.02 | 36.70 | 103.55 | 32.73 | 95.78 | 33.62 | 91.37 | 31.56 | 85.25 | 31.99 | 77.99 | 32.33 | 68.55 | 31.53 | 62.70 | 30.99 | 58.44 | 28.53 | 53.94 | 28.21 | |
| eGFR (CKD-EPI)) | |  |  |  |  |  |  |  |  |  |  |  |  |  |  |  |  |  |  |  |  |  | |
| 2006 | 113.79 | 29.81 | 101.54 | 37.20 | 99.47 | 34.90 | 94.63 | 34.44 | 85.91 | 35.79 | 79.60 | 31.88 | 71.72 | 32.44 | 63.27 | 32.60 | 58.10 | 31.51 | 51.31 | 30.44 | 46.69 | 26.98 | |
| 2007 | 114.29 | 32.43 | 104.99 | 31.52 | 95.32 | 35.04 | 97.55 | 28.40 | 88.23 | 31.43 | 79.60 | 31.26 | 70.74 | 30.42 | 63.87 | 30.37 | 57.31 | 30.26 | 50.74 | 28.79 | 46.38 | 25.25 | |
| 2008 | 112.55 | 35.88 | 105.92 | 34.16 | 101.60 | 33.06 | 99.32 | 28.22 | 92.66 | 29.19 | 81.37 | 32.87 | 72.53 | 33.47 | 65.11 | 31.50 | 59.80 | 29.52 | 52.82 | 29.27 | 48.78 | 27.60 | |
| 2009 | 104.77 | 37.66 | 104.79 | 34.38 | 99.55 | 33.72 | 95.89 | 30.85 | 89.94 | 29.89 | 80.45 | 32.30 | 69.58 | 32.95 | 63.32 | 32.10 | 56.14 | 29.21 | 52.97 | 28.90 | 46.68 | 26.53 | |
| 2010 | 113.76 | 34.59 | 110.16 | 30.32 | 101.82 | 32.11 | 97.41 | 31.77 | 90.84 | 30.57 | 83.11 | 32.03 | 72.14 | 33.03 | 66.07 | 31.51 | 58.88 | 29.89 | 55.72 | 27.71 | 48.75 | 26.30 | |
| 2011 | 114.93 | 35.81 | 107.71 | 33.97 | 102.41 | 33.15 | 97.75 | 33.01 | 94.52 | 29.28 | 85.37 | 31.49 | 76.81 | 31.95 | 68.05 | 32.00 | 62.66 | 29.17 | 55.20 | 28.66 | 49.03 | 26.14 | |
| 2012 | 116.92 | 30.01 | 109.05 | 31.54 | 105.89 | 28.98 | 101.24 | 26.72 | 92.80 | 29.19 | 84.87 | 31.37 | 77.64 | 31.19 | 67.05 | 30.94 | 62.25 | 29.20 | 55.73 | 28.26 | 49.83 | 25.46 | |
| 2013 | 117.51 | 30.52 | 111.80 | 29.78 | 105.97 | 29.13 | 102.66 | 25.81 | 94.60 | 27.73 | 87.38 | 29.26 | 79.02 | 29.79 | 70.36 | 29.90 | 63.01 | 29.02 | 58.88 | 27.43 | 52.04 | 25.08 | |
| 2014 | 115.98 | 29.94 | 111.66 | 28.24 | 104.39 | 30.68 | 101.51 | 26.55 | 94.57 | 27.23 | 87.22 | 29.16 | 79.55 | 29.68 | 69.25 | 30.07 | 62.65 | 28.80 | 57.60 | 27.31 | 51.98 | 25.02 | |
| 2015 | 115.88 | 31.64 | 112.10 | 28.51 | 107.96 | 26.25 | 100.37 | 28.61 | 95.71 | 26.99 | 88.93 | 27.87 | 80.87 | 29.30 | 71.13 | 29.93 | 64.16 | 29.18 | 59.21 | 27.12 | 52.54 | 25.23 | |

CKD-EPI: Chronic Kidney Disease Epidemiology Collaboration; eGFR: estimated glomerular filtration rate; MDRD: Modification of diet in renal disease Y: Years
