# Supplementary material for: Renal insufficiency among urban populations in Bangladesh: A decade of laboratory-based observations
Source: PLoS One. 2019 Apr 4;14(4):e0214568. doi: 10.1371/journal.pone.0214568 (PMC6448896; doi:10.1371/journal.pone.0214568)
Supplement: S3 Table — (DOCX) [file pone.0214568.s003.docx]

**S3 Table:** Overall and sex-specific yearly distribution of renal insufficiency stages following MDRD and abbreviated MDRD definition

|  | MDRD | | | | | | | | | |  | Abbreviated MDRD | | | | | | | | | | |
| --- | --- | --- | --- | --- | --- | --- | --- | --- | --- | --- | --- | --- | --- | --- | --- | --- | --- | --- | --- | --- | --- | --- |
| Year | Stage1 | | Stage2 | | Stage3 | | Stage4 | | Stage5 | |  | Stage1 | | Stage2 | | Stage3 | | Stage4 | | Stage5 | | |
|  | % | n | % | n | % | n | % | n | % | n |  | % | n | % | n | % | n | % | n | % | n |  |
| 2006 | 36.9 | 3,071 | 25.3 | 2,108 | 19.8 | 1,648 | 9.13 | 760 | 8.8 | 733 |  | 31.09 | 2,587 | 28.45 | 2,367 | 21.42 | 1,782 | 9.63 | 801 | 9.41 | 783 |  |
| 2007 | 34.1 | 3,378 | 29.6 | 2,931 | 20.1 | 1,985 | 9.42 | 932 | 6.8 | 670 |  | 28.44 | 2,814 | 32.44 | 3,210 | 21.66 | 2,143 | 10.23 | 1,012 | 7.25 | 717 |  |
| 2008 | 39.9 | 4,759 | 26.2 | 3,121 | 17.9 | 2,136 | 9.08 | 1,083 | 7 | 833 |  | 33.65 | 4,015 | 30.13 | 3,595 | 19.00 | 2,267 | 9.60 | 1,145 | 7.63 | 910 |  |
| 2009 | 36.3 | 5,372 | 27.5 | 4,068 | 19.6 | 2,907 | 9.35 | 1,384 | 7.2 | 1,070 |  | 30.75 | 4,551 | 30.68 | 4,541 | 20.70 | 3,064 | 10.12 | 1,498 | 7.75 | 1,147 |  |
| 2010 | 40.1 | 6,907 | 25.6 | 4,399 | 19.3 | 3,326 | 8.72 | 1,501 | 6.3 | 1,081 |  | 34.14 | 5,877 | 29.14 | 5,017 | 20.49 | 3,528 | 9.45 | 1,627 | 6.77 | 1,165 |  |
| 2011 | 43 | 9,354 | 25.3 | 5,491 | 18.2 | 3,960 | 7.74 | 1,682 | 5.8 | 1,252 |  | 37.07 | 8,058 | 29.02 | 6,309 | 19.24 | 4,183 | 8.52 | 1,853 | 6.15 | 1,336 |  |
| 2012 | 42.1 | 11,290 | 27.2 | 7,300 | 17.9 | 4,800 | 7.39 | 1,984 | 5.4 | 1,456 |  | 35.78 | 9,601 | 30.92 | 8,297 | 19.46 | 5,221 | 8.05 | 2,159 | 5.78 | 1,552 |  |
| 2013 | 44.1 | 13,307 | 27.5 | 8,307 | 17.4 | 5,263 | 6.74 | 2,036 | 4.3 | 1,290 |  | 37.82 | 11,423 | 31.28 | 9,448 | 18.87 | 5,698 | 7.44 | 2,247 | 4.59 | 1,387 |  |
| 2014 | 42.7 | 15,710 | 28.2 | 10,373 | 17.8 | 6,557 | 6.72 | 2,476 | 4.6 | 1,705 |  | 36.18 | 13,320 | 32.08 | 11,812 | 19.37 | 7,134 | 7.40 | 2,725 | 4.97 | 1,830 |  |
| 2015 | 44.2 | 18,189 | 28.2 | 11,581 | 17.1 | 7,029 | 6.14 | 2,526 | 4.4 | 1,807 |  | 37.66 | 15,489 | 32.29 | 13,283 | 18.73 | 7,702 | 6.64 | 2,730 | 4.69 | 1,928 |  |
| Male |  |  |  |  |  |  |  |  |  |  |  |  |  |  |  |  |  |  |  |  |  |  |
| 2006 | 33.1 | 1,560 | 30.4 | 1,433 | 19.9 | 935 | 7.75 | 365 | 8.9 | 417 |  | 26.86 | 1,265 | 33.63 | 1,584 | 21.89 | 1,031 | 8.32 | 392 | 9.30 | 438 |  |
| 2007 | 31.3 | 1,732 | 34.5 | 1,910 | 19.9 | 1,099 | 8.04 | 445 | 6.3 | 348 |  | 25.10 | 1,389 | 37.37 | 2,068 | 21.96 | 1,215 | 8.80 | 487 | 6.78 | 375 |  |
| 2008 | 36.1 | 2,401 | 32.3 | 2,148 | 17.8 | 1,184 | 7.74 | 514 | 6 | 396 |  | 29.05 | 1,930 | 36.49 | 2,424 | 19.83 | 1,317 | 8.08 | 537 | 6.55 | 435 |  |
| 2009 | 31.9 | 2,653 | 32.9 | 2,733 | 20.2 | 1,680 | 8.16 | 678 | 6.8 | 566 |  | 25.80 | 2,144 | 36.19 | 3,007 | 21.90 | 1,820 | 8.76 | 728 | 7.35 | 611 |  |
| 2010 | 35.6 | 3,413 | 31 | 2,969 | 20 | 1,920 | 7.47 | 716 | 6 | 572 |  | 28.55 | 2,738 | 35.09 | 3,365 | 21.87 | 2,097 | 8.12 | 779 | 6.37 | 611 |  |
| 2011 | 40 | 5,019 | 30.1 | 3,782 | 18.5 | 2,325 | 6.44 | 809 | 4.9 | 618 |  | 33.26 | 4,175 | 34.29 | 4,304 | 20.04 | 2,515 | 7.21 | 905 | 5.21 | 654 |  |
| 2012 | 39.3 | 5,943 | 32.9 | 4,977 | 17.7 | 2,681 | 6.14 | 929 | 4 | 610 |  | 31.82 | 4,817 | 37.25 | 5,639 | 19.91 | 3,015 | 6.76 | 1,023 | 4.27 | 646 |  |
| 2013 | 40.9 | 7,072 | 33.3 | 5,755 | 17.2 | 2,970 | 5.37 | 928 | 3.2 | 550 |  | 33.75 | 5,831 | 37.56 | 6,488 | 19.28 | 3,331 | 5.95 | 1,027 | 3.46 | 598 |  |
| 2014 | 39.4 | 8,231 | 34.2 | 7,153 | 17.6 | 3,684 | 5.45 | 1,140 | 3.4 | 711 |  | 31.90 | 6,673 | 38.54 | 8,062 | 19.88 | 4,158 | 6.04 | 1,263 | 3.65 | 763 |  |
| 2015 | 41 | 9,545 | 34.2 | 7,950 | 16.7 | 3,876 | 4.84 | 1,128 | 3.4 | 784 |  | 33.49 | 7,797 | 38.81 | 9,037 | 18.80 | 4,378 | 5.31 | 1,236 | 3.59 | 835 |  |
| Female | |  |  |  |  |  |  |  |  |  |  |  |  |  |  |  |  |  |  |  |  |  |
| 2006 | 41.9 | 1,511 | 18.7 | 675 | 19.8 | 713 | 10.9 | 395 | 8.8 | 316 |  | 36.62 | 1,322 | 21.69 | 783 | 20.80 | 751 | 11.33 | 409 | 9.56 | 345 |  |
| 2007 | 37.7 | 1,646 | 23.4 | 1,021 | 20.3 | 886 | 11.2 | 487 | 7.4 | 322 |  | 32.67 | 1,425 | 26.18 | 1,142 | 21.27 | 928 | 12.04 | 525 | 7.84 | 342 |  |
| 2008 | 44.6 | 2,358 | 18.4 | 973 | 18 | 952 | 10.8 | 569 | 8.3 | 437 |  | 39.42 | 2,085 | 22.14 | 1,171 | 17.96 | 950 | 11.50 | 608 | 8.98 | 475 |  |
| 2009 | 41.9 | 2,719 | 20.6 | 1,335 | 18.9 | 1,227 | 10.9 | 706 | 7.8 | 504 |  | 37.08 | 2,407 | 23.63 | 1,534 | 19.16 | 1,244 | 11.86 | 770 | 8.26 | 536 |  |
| 2010 | 45.8 | 3,494 | 18.8 | 1,430 | 18.4 | 1,406 | 10.3 | 785 | 6.7 | 509 |  | 41.17 | 3,139 | 21.67 | 1,652 | 18.77 | 1,431 | 11.12 | 848 | 7.27 | 554 |  |
| 2011 | 47.2 | 4,335 | 18.6 | 1,709 | 17.8 | 1,635 | 9.5 | 873 | 6.9 | 634 |  | 42.27 | 3,883 | 21.83 | 2,005 | 18.16 | 1,668 | 10.32 | 948 | 7.42 | 682 |  |
| 2012 | 45.7 | 5,347 | 19.9 | 2,323 | 18.1 | 2,119 | 9.02 | 1,055 | 7.2 | 846 |  | 40.92 | 4,784 | 22.74 | 2,658 | 18.87 | 2,206 | 9.72 | 1,136 | 7.75 | 906 |  |
| 2013 | 48.2 | 6,235 | 19.7 | 2,552 | 17.7 | 2,293 | 8.57 | 1,108 | 5.7 | 740 |  | 43.25 | 5,592 | 22.90 | 2,960 | 18.31 | 2,367 | 9.44 | 1,220 | 6.10 | 789 |  |
| 2014 | 47 | 7,479 | 20.3 | 3,220 | 18.1 | 2,873 | 8.4 | 1,336 | 6.3 | 994 |  | 41.80 | 6,647 | 23.58 | 3,750 | 18.71 | 2,976 | 9.19 | 1,462 | 6.71 | 1,067 |  |
| 2015 | 48.4 | 8,644 | 20.3 | 3,631 | 17.7 | 3,153 | 7.83 | 1,398 | 5.7 | 1,023 |  | 43.09 | 7,692 | 23.79 | 4,246 | 18.62 | 3,324 | 8.37 | 1,494 | 6.12 | 1,093 |  |

MDRD: Modification of diet in renal disease
