# Supplementary material for: Renal insufficiency among urban populations in Bangladesh: A decade of laboratory-based observations
Source: PLoS One. 2019 Apr 4;14(4):e0214568. doi: 10.1371/journal.pone.0214568 (PMC6448896; doi:10.1371/journal.pone.0214568)
Supplement: S4 Table — (DOCX) [file pone.0214568.s004.docx]

**S4 Table:** Age-stratified prevalence of renal insufficiency stages following MDRD and abbreviated MDRD equations

|  | MDRD definition | | | | | | | | | |  | Abbreviated MDRD | | | | | | | | | |
| --- | --- | --- | --- | --- | --- | --- | --- | --- | --- | --- | --- | --- | --- | --- | --- | --- | --- | --- | --- | --- | --- |
| Age  stratum | Stage1 | | Stage2 | | Stage3 | | Stage4 | | Stage5 | |  | Stage1 | | Stage2 | | Stage3 | | Stage4 | | Stage5 | |
|  | % | n | % | n | % | n | % | n | % | n |  | % | n | % | n | % | n | % | n | % | n |
| 19-24Y | 81 | 3,950 | 8.15 | 397 | 6.46 | 315 | 1.89 | 92 | 2.46 | 120 |  | 78.6 | 3,831 | 9.66 | 471 | 7.14 | 348 | 2.05 | 100 | 2.54 | 124 |
| 24-28Y | 78.4 | 7,171 | 10.4 | 952 | 6.45 | 590 | 2.8 | 256 | 2 | 183 |  | 74.3 | 6,800 | 13.5 | 1,234 | 7.09 | 649 | 2.97 | 272 | 2.15 | 197 |
| 29-33Y | 74.2 | 9,372 | 13.8 | 1,746 | 6.28 | 793 | 2.89 | 365 | 2.76 | 349 |  | 68.3 | 8,620 | 19 | 2,393 | 6.78 | 856 | 3.03 | 383 | 2.95 | 373 |
| 34-38Y | 70.4 | 12,233 | 17.5 | 3,046 | 6.73 | 1,170 | 2.72 | 473 | 2.64 | 459 |  | 62.7 | 10,892 | 24.4 | 4,240 | 7.23 | 1,256 | 2.9 | 504 | 2.81 | 489 |
| 39-43Y | 61.4 | 13,670 | 23.6 | 5,241 | 8.85 | 1,969 | 3.42 | 761 | 2.77 | 616 |  | 53.1 | 11,828 | 30.7 | 6,828 | 9.53 | 2,120 | 3.71 | 826 | 2.94 | 655 |
| 44-48Y | 51.6 | 13,638 | 27.3 | 7,219 | 12.4 | 3,268 | 4.85 | 1,281 | 3.83 | 1,011 |  | 43.5 | 11,495 | 33.7 | 8,897 | 13.4 | 3,541 | 5.33 | 1,408 | 4.07 | 1,076 |
| 49-53Y | 40.4 | 11,443 | 31.2 | 8,846 | 16.2 | 4,576 | 6.9 | 1,955 | 5.3 | 1,500 |  | 32.7 | 9,270 | 36.6 | 10,366 | 17.4 | 4,925 | 7.57 | 2,143 | 5.71 | 1,616 |
| 54-58Y | 29 | 8,459 | 33.8 | 9,862 | 21.1 | 6,166 | 8.94 | 2,609 | 7.17 | 2,092 |  | 22.5 | 6,576 | 37.2 | 10,865 | 22.9 | 6,691 | 9.7 | 2,832 | 7.62 | 2,224 |
| 59-63Y | 21.4 | 5,632 | 34.1 | 8,980 | 26.1 | 6,852 | 11.2 | 2,950 | 7.19 | 1,891 |  | 16.1 | 4,232 | 35.9 | 9,437 | 28.2 | 7,404 | 12.1 | 3,179 | 7.8 | 2,053 |
| 64-68Y | 16.5 | 3,065 | 32.8 | 6,103 | 30.5 | 5,678 | 11.7 | 2,174 | 8.61 | 1,603 |  | 11.8 | 2,202 | 33.3 | 6,207 | 32.8 | 6,103 | 12.8 | 2,390 | 9.24 | 1,721 |
| ≥69Y | 11.4 | 2,704 | 30.7 | 7,287 | 34.7 | 8,234 | 14.5 | 3,448 | 8.73 | 2,073 |  | 8.38 | 1,989 | 29.2 | 6,941 | 37.2 | 8,829 | 15.8 | 3,760 | 9.38 | 2,227 |
| **Male** |  |  |  |  |  |  |  |  |  |  |  |  |  |  |  |  |  |  |  |  |  |
| 19-24Y | 75 | 1,789 | 11.3 | 270 | 9.68 | 231 | 1.93 | 46 | 2.14 | 51 |  | 72.1 | 1,721 | 12.9 | 307 | 10.7 | 255 | 2.14 | 51 | 2.22 | 53 |
| 24-28Y | 73 | 3,397 | 13.5 | 626 | 7.54 | 351 | 3.33 | 155 | 2.71 | 126 |  | 67.3 | 3,134 | 18 | 838 | 8.21 | 382 | 3.67 | 171 | 2.79 | 130 |
| 29-33Y | 67.8 | 4,829 | 17.5 | 1,249 | 7.72 | 550 | 3.42 | 244 | 3.59 | 256 |  | 60.1 | 4,284 | 24.1 | 1,720 | 8.29 | 591 | 3.68 | 262 | 3.8 | 271 |
| 34-38Y | 65.6 | 6,436 | 22 | 2,164 | 7 | 687 | 2.43 | 239 | 2.96 | 291 |  | 56.2 | 5,515 | 30.4 | 2,988 | 7.69 | 755 | 2.53 | 248 | 3.17 | 311 |
| 39-43Y | 57.3 | 7,401 | 28.8 | 3,713 | 8.62 | 1,113 | 2.77 | 358 | 2.56 | 330 |  | 47.6 | 6,141 | 37.2 | 4,806 | 9.51 | 1,228 | 3.02 | 390 | 2.71 | 350 |
| 44-48Y | 48 | 6,945 | 33.6 | 4,869 | 11.9 | 1,726 | 3.65 | 529 | 2.8 | 405 |  | 38.5 | 5,570 | 41 | 5,940 | 13.4 | 1,939 | 4.12 | 597 | 2.96 | 428 |
| 49-53Y | 39.1 | 5,929 | 38.1 | 5,779 | 14.1 | 2,143 | 4.93 | 748 | 3.82 | 580 |  | 30.1 | 4,564 | 44.4 | 6,737 | 16 | 2,423 | 5.52 | 838 | 4.06 | 617 |
| 54-58Y | 27.9 | 4,431 | 41.3 | 6,563 | 19.8 | 3,142 | 6.13 | 973 | 4.84 | 768 |  | 20.7 | 3,280 | 44.8 | 7,112 | 22.7 | 3,597 | 6.75 | 1,072 | 5.14 | 816 |
| 59-63Y | 19.5 | 3,019 | 39.8 | 6,151 | 24.8 | 3,832 | 9.92 | 1,534 | 6 | 928 |  | 13.8 | 2,138 | 41.3 | 6,380 | 27.8 | 4,302 | 10.5 | 1,627 | 6.58 | 1,017 |
| 64-68Y | 15.5 | 1,698 | 37.5 | 4,100 | 30.9 | 3,375 | 9.18 | 1,004 | 6.97 | 763 |  | 10.8 | 1,180 | 37.6 | 4,110 | 34 | 3,715 | 10.3 | 1,122 | 7.43 | 813 |
| ≥69Y | 11.2 | 1,695 | 35.2 | 5,326 | 34.4 | 5,204 | 12.1 | 1,822 | 7.1 | 1,074 |  | 8.15 | 1,232 | 33.3 | 5,040 | 37.6 | 5,690 | 13.2 | 1,999 | 7.67 | 1,160 |
| **Female** |  |  |  |  |  |  |  |  |  |  |  |  |  |  |  |  |  |  |  |  |  |
| 19-24Y | 86.9 | 2,161 | 5.11 | 127 | 3.38 | 84 | 1.85 | 46 | 2.77 | 69 |  | 84.8 | 2,110 | 6.59 | 164 | 3.74 | 93 | 1.97 | 49 | 2.85 | 71 |
| 24-28Y | 83.9 | 3,774 | 7.25 | 326 | 5.31 | 239 | 2.25 | 101 | 1.27 | 57 |  | 81.5 | 3,666 | 8.81 | 396 | 5.94 | 267 | 2.25 | 101 | 1.49 | 67 |
| 29-33Y | 82.7 | 4,543 | 9.04 | 497 | 4.42 | 243 | 2.2 | 121 | 1.69 | 93 |  | 78.9 | 4,336 | 12.2 | 673 | 4.82 | 265 | 2.2 | 121 | 1.86 | 102 |
| 34-38Y | 76.6 | 5,797 | 11.7 | 882 | 6.39 | 483 | 3.09 | 234 | 2.22 | 168 |  | 71.1 | 5,377 | 16.6 | 1,252 | 6.62 | 501 | 3.38 | 256 | 2.35 | 178 |
| 39-43Y | 67.1 | 6,269 | 16.4 | 1,528 | 9.16 | 856 | 4.31 | 403 | 3.06 | 286 |  | 60.9 | 5,687 | 21.6 | 2,022 | 9.55 | 892 | 4.67 | 436 | 3.26 | 305 |
| 44-48Y | 56 | 6,693 | 19.7 | 2,350 | 12.9 | 1,542 | 6.3 | 752 | 5.07 | 606 |  | 49.6 | 5,925 | 24.8 | 2,957 | 13.4 | 1,602 | 6.79 | 811 | 5.43 | 648 |
| 49-53Y | 42 | 5,514 | 23.3 | 3,067 | 18.5 | 2,433 | 9.18 | 1,207 | 7 | 920 |  | 35.8 | 4,706 | 27.6 | 3,629 | 19 | 2,502 | 9.93 | 1,305 | 7.6 | 999 |
| 54-58Y | 30.3 | 4,028 | 24.8 | 3,299 | 22.7 | 3,024 | 12.3 | 1,636 | 9.95 | 1,324 |  | 24.8 | 3,296 | 28.2 | 3,753 | 23.2 | 3,094 | 13.2 | 1,760 | 10.6 | 1,408 |
| 59-63Y | 24.1 | 2,613 | 26.1 | 2,829 | 27.9 | 3,020 | 13.1 | 1,416 | 8.88 | 963 |  | 19.3 | 2,094 | 28.2 | 3,057 | 28.6 | 3,102 | 14.3 | 1,552 | 9.56 | 1,036 |
| 64-68Y | 17.8 | 1,367 | 26.1 | 2,003 | 30 | 2,303 | 15.2 | 1,170 | 10.9 | 840 |  | 13.3 | 1,022 | 27.3 | 2,097 | 31.1 | 2,388 | 16.5 | 1,268 | 11.8 | 908 |
| ≥69Y | 11.7 | 1,009 | 22.7 | 1,961 | 35.1 | 3,030 | 18.9 | 1,626 | 11.6 | 999 |  | 8.78 | 757 | 22 | 1,901 | 36.4 | 3,139 | 20.4 | 1,761 | 12.4 | 1,067 |

MDRD: Modification of diet in renal disease Y: Years
