# Supplementary material for: Renal insufficiency among urban populations in Bangladesh: A decade of laboratory-based observations
Source: PLoS One. 2019 Apr 4;14(4):e0214568. doi: 10.1371/journal.pone.0214568 (PMC6448896; doi:10.1371/journal.pone.0214568)
Supplement: S5 Table — (DOCX) [file pone.0214568.s005.docx]

**S5 Table:** Distribution of renal insufficiency stages in different age groups by MDRD and abbreviated MDRD equations in 2006 and 2015

|  |  |  | **MDRD definition** | | | | | | | | | | | | | |  | | **Abbreviated MDRD** | | | | | | | | | | | | | | |
| --- | --- | --- | --- | --- | --- | --- | --- | --- | --- | --- | --- | --- | --- | --- | --- | --- | --- | --- | --- | --- | --- | --- | --- | --- | --- | --- | --- | --- | --- | --- | --- | --- | --- |
|  |  |  | **Stage2** | | **Stage3** | | **Stage4** | | **Stage5** | | **Overall** | **Stag2** | **Stag3** | **Stag4** | **Stag5** |  | | **Stage2** | | | **Stage3** | | | **Stage4** | | | **Stage5** | | **Overall** | **Stag2** | **Stag3** | **Stag4** | **Stag5** |
| **Age group** | **Year** | **N** | **n** | **%** | **n** | **%** | **n** | **%** | **n** | **%** |  |  |  |  |  |  | | **n** | | **%** | **n** | **%** | **n** | | **%** | **n** | | **%** |  |  |  |  |  |
| 19-24Y | 2006 | 171 | 22 | 12.9 | 10 | 5.85 | 2 | 1.17 | 2 | 1.17 | 4.68 | 2.45 | 5.20 | 4.00 | 10.50 |  | | 26 | | 15.2 | 12 | 7.02 | 2 | | 1.17 | 2 | | 1.17 | 4.68 | 2.54 | 4.75 | 4.50 | 10.50 |
|  | 2015 | 801 | 54 | 6.74 | 52 | 6.49 | 8 | 1 | 21 | 2.62 |  |  |  |  |  |  | | 66 | | 8.24 | 57 | 7.12 | 9 | | 1.12 | 21 | | 2.62 |  |  |  |  |  |
| 24-28Y | 2006 | 333 | 36 | 10.8 | 39 | 11.7 | 20 | 6.01 | 8 | 2.4 | 4.76 | 4.50 | 1.92 | 1.45 | 3.50 |  | | 43 | | 12.9 | 43 | 12.9 | 19 | | 5.71 | 10 | | 3.0 | 4.76 | 5.28 | 1.86 | 1.68 | 2.80 |
|  | 2015 | 1,586 | 162 | 10.2 | 75 | 4.73 | 29 | 1.83 | 28 | 1.77 |  |  |  |  |  |  | | 227 | | 14.3 | 80 | 5.04 | 32 | | 2.02 | 28 | | 1.77 |  |  |  |  |  |
| 29-33Y | 2006 | 448 | 69 | 15.4 | 42 | 9.38 | 14 | 3.13 | 19 | 4.24 | 5.17 | 4.55 | 2.07 | 3.14 | 2.26 |  | | 83 | | 18.5 | 44 | 9.82 | 16 | | 3.57 | 20 | | 4.46 | 5.17 | 5.37 | 2.27 | 2.88 | 2.15 |
|  | 2015 | 2,317 | 314 | 13.6 | 87 | 3.75 | 44 | 1.9 | 43 | 1.86 |  |  |  |  |  |  | | 446 | | 19.3 | 100 | 4.32 | 46 | | 1.99 | 43 | | 1.86 |  |  |  |  |  |
| 34-38Y | 2006 | 677 | 105 | 15.5 | 66 | 9.75 | 21 | 3.1 | 37 | 5.47 | 4.93 | 5.34 | 3.00 | 4.14 | 2.54 |  | | 151 | | 22.3 | 67 | 9.9 | 26 | | 3.84 | 40 | | 5.91 | 4.93 | 5.38 | 3.12 | 3.65 | 2.45 |
|  | 2015 | 3,339 | 561 | 16.8 | 198 | 5.93 | 87 | 2.61 | 94 | 2.82 |  |  |  |  |  |  | | 813 | | 24.4 | 209 | 6.26 | 95 | | 2.85 | 98 | | 2.94 |  |  |  |  |  |
| 39-43Y | 2006 | 793 | 150 | 18.9 | 84 | 10.6 | 59 | 7.44 | 52 | 6.56 | 5.26 | 6.41 | 3.67 | 1.97 | 1.73 |  | | 208 | | 26.2 | 92 | 11.6 | 57 | | 7.19 | 55 | | 6.94 | 5.26 | 6.26 | 3.62 | 2.25 | 1.75 |
|  | 2015 | 4,171 | 961 | 23.0 | 308 | 7.38 | 116 | 2.78 | 90 | 2.16 |  |  |  |  |  |  | | 1,303 | | 31.2 | 333 | 7.98 | 128 | | 3.07 | 96 | | 2.3 |  |  |  |  |  |
| 44-48Y | 2006 | 881 | 253 | 28.7 | 157 | 17.8 | 43 | 4.88 | 48 | 5.45 | 5.96 | 5.91 | 3.38 | 4.30 | 2.98 |  | | 282 | | 32.0 | 185 | 21 | 43 | | 4.88 | 53 | | 6.02 | 5.96 | 6.63 | 3.20 | 4.93 | 2.77 |
|  | 2015 | 5,248 | 1,496 | 28.5 | 531 | 10.1 | 185 | 3.53 | 143 | 2.72 |  |  |  |  |  |  | | 1,871 | | 35.7 | 592 | 11.3 | 212 | | 4.04 | 147 | | 2.8 |  |  |  |  |  |
| 49-53Y | 2006 | 1,225 | 362 | 29.6 | 235 | 19.2 | 101 | 8.24 | 95 | 7.76 | 4.25 | 4.50 | 3.36 | 2.58 | 2.01 |  | | 417 | | 34.0 | 262 | 21.4 | 100 | | 8.16 | 107 | | 8.73 | 4.25 | 4.53 | 3.31 | 2.89 | 1.91 |
|  | 2015 | 5,206 | 1,628 | 31.3 | 790 | 15.2 | 261 | 5.01 | 191 | 3.67 |  |  |  |  |  |  | | 1,889 | | 36.3 | 866 | 16.6 | 289 | | 5.55 | 204 | | 3.92 |  |  |  |  |  |
| 54-58Y | 2006 | 1,138 | 343 | 30.1 | 267 | 23.5 | 122 | 10.7 | 119 | 10.5 | 4.57 | 5.26 | 3.77 | 3.34 | 2.47 |  | | 373 | | 32.8 | 283 | 24.9 | 131 | | 11.5 | 125 | | 11.0 | 4.57 | 5.45 | 3.87 | 3.31 | 2.54 |
|  | 2015 | 5,196 | 1,805 | 34.7 | 1,007 | 19.4 | 408 | 7.85 | 294 | 5.66 |  |  |  |  |  |  | | 2,033 | | 39.1 | 1,096 | 21.1 | 434 | | 8.35 | 317 | | 6.1 |  |  |  |  |  |
| 59-63Y | 2006 | 952 | 307 | 32.3 | 218 | 22.9 | 141 | 14.8 | 96 | 10.1 | 5.32 | 5.83 | 5.78 | 3.33 | 3.59 |  | | 313 | | 32.9 | 243 | 25.5 | 142 | | 14.9 | 104 | | 10.9 | 5.32 | 6.03 | 5.73 | 3.48 | 3.56 |
|  | 2015 | 5,069 | 1,790 | 35.3 | 1,261 | 24.9 | 470 | 9.27 | 345 | 6.81 |  |  |  |  |  |  | | 1,887 | | 37.2 | 1,392 | 27.5 | 494 | | 9.75 | 370 | | 7.3 |  |  |  |  |  |
| 64-68Y | 2006 | 782 | 215 | 27.5 | 216 | 27.6 | 104 | 13.3 | 121 | 15.5 | 4.71 | 6.00 | 5.22 | 3.56 | 1.90 |  | | 229 | | 29.3 | 226 | 28.9 | 118 | | 15.1 | 126 | | 16.1 | 4.71 | 5.71 | 5.50 | 3.42 | 1.98 |
|  | 2015 | 3,680 | 1,289 | 35.0 | 1,128 | 30.7 | 370 | 10.1 | 230 | 6.25 |  |  |  |  |  |  | | 1,308 | | 35.5 | 1,243 | 33.8 | 404 | | 11.0 | 249 | | 6.77 |  |  |  |  |  |
| ≥69Y | 2006 | 920 | 246 | 26.7 | 314 | 34.1 | 133 | 14.5 | 136 | 14.8 | 4.91 | 6.18 | 5.07 | 4.12 | 2.41 |  | | 242 | | 26.3 | 325 | 35.3 | 147 | | 16.0 | 141 | | 15.3 | 4.91 | 5.95 | 5.34 | 3.99 | 2.52 |
|  | 2015 | 4,519 | 1,521 | 33.7 | 1,592 | 35.2 | 548 | 12.1 | 328 | 7.26 |  |  |  |  |  |  | | 1440 | | 31.9 | 1,734 | 38.4 | 587 | | 13.0 | 355 | | 7.86 |  |  |  |  |  |
| *ratio of number of CKD stage 2-5 between 2015 and 2006 | | | | | | | | | | | | | | | | | | | | | | | | | | | | | | | | | |

MDRD: Modification of diet in renal disease Y: Years
