# Supplementary material for: Renal insufficiency among urban populations in Bangladesh: A decade of laboratory-based observations
Source: PLoS One. 2019 Apr 4;14(4):e0214568. doi: 10.1371/journal.pone.0214568 (PMC6448896; doi:10.1371/journal.pone.0214568)
Supplement: S6 Table — (DOCX) [file pone.0214568.s006.docx]

**S6 Table:** Overall and sex-specific association of eGFR following MDRD and Abbreviated MDRD equations

|  | Coef. | 95% CI | |
| --- | --- | --- | --- |
| **MDRD** |  | LL | UL |
| Overall (N=218888) | -1.29 | -1.30 | -1.28 |
| Male (N=123957) | -1.05 | -1.07 | -1.04 |
| Female (N=94931) | -1.62 | -1.64 | -1.60 |
| ***Adjusted for blood glucose*** | |  |  |
| Overall (N=30582) | -1.31 | -1.34 | -1.29 |
| Male (N=18165) | -1.07 | -1.10 | -1.05 |
| Female (N=12417) | -1.67 | -1.71 | -1.62 |
| ***Abbreviated MDRD*** | |  |  |
| Overall (N=218888) | -1.22 | -1.23 | -1.21 |
| Male (N=123957) | -0.99 | -1.00 | -0.98 |
| Female (N=94931) | -1.53 | -1.54 | -1.51 |
| ***Adjusted for blood glucose*** | |  |  |
| Overall (N=30582) | -1.23 | -1.25 | -1.21 |
| Male (N=18165) | -1.01 | -1.04 | -0.99 |
| Female (N=12417) | -1.57 | -1.61 | -1.52 |

NB: Stage 1 considered as reference group; MDRD: Modification of diet in renal disease
