# Supplementary material for: Renal insufficiency among urban populations in Bangladesh: A decade of laboratory-based observations
Source: PLoS One. 2019 Apr 4;14(4):e0214568. doi: 10.1371/journal.pone.0214568 (PMC6448896; doi:10.1371/journal.pone.0214568)
Supplement: S7 Table — (DOCX) [file pone.0214568.s007.docx]

**S7 Table**: Overall and sex-specific adjusted risk of renal insufficiency following MDRD and Abbreviated MDRD equations

|  | MDRD | | | | | | | | | | | |  | | Abbreviated MDRD | | | | | | | | | | | | | | |
| --- | --- | --- | --- | --- | --- | --- | --- | --- | --- | --- | --- | --- | --- | --- | --- | --- | --- | --- | --- | --- | --- | --- | --- | --- | --- | --- | --- | --- | --- |
|  | Overall (N=218888) | | |  | Male (N=123957) | | |  | Female (N=94931) | | | |  | Overall (N=218888) | | | | |  | | Male (N=123957) | | | |  | | Female (N=94931) | | |
|  | OR | 95% Conf. | |  | OR | 95% Conf. | |  | OR | 95% Conf. | |  | | OR | | 95% Conf. | | |  | | OR | 95% Conf. | | |  | | OR | 95% Conf. | |
|  |  | LL | UL |  |  | LL | UL |  |  | LL | UL |  | |  | | LL | UL |  | |  | | LL | UL |  | |  | | LL | UL |
| Stage 2 |  |  |  |  |  |  |  |  |  |  |  |  | |  | |  |  |  | |  | |  |  |  | |  | |  |  |
| 19-45Y | Ref. | Ref. | Ref. |  | Ref. | Ref. | Ref. |  | Ref. | Ref. | Ref. |  | | Ref. | | Ref. | Ref. |  | | Ref. | | Ref. | Ref. |  | | Ref. | | Ref. | Ref. |
| 45-48Y | 2.16 | 2.08 | 2.24 |  | 2.05 | 1.96 | 2.15 |  | 2.33 | 2.19 | 2.48 |  | | 2.14 | | 2.07 | 2.22 |  | | 2.04 | | 1.95 | 2.14 |  | | 2.28 | | 2.15 | 2.41 |
| 49-53Y | 3.12 | 3.01 | 3.23 |  | 2.79 | 2.67 | 2.91 |  | 3.60 | 3.41 | 3.81 |  | | 3.09 | | 2.99 | 3.20 |  | | 2.77 | | 2.65 | 2.90 |  | | 3.48 | | 3.30 | 3.67 |
| 54-58Y | 4.70 | 4.54 | 4.87 |  | 4.24 | 4.05 | 4.44 |  | 5.32 | 5.02 | 5.64 |  | | 4.57 | | 4.41 | 4.73 |  | | 4.08 | | 3.89 | 4.27 |  | | 5.16 | | 4.87 | 5.45 |
| 59-63Y | 6.27 | 6.03 | 6.52 |  | 5.86 | 5.58 | 6.17 |  | 7.05 | 6.62 | 7.52 |  | | 5.98 | | 5.74 | 6.23 |  | | 5.64 | | 5.34 | 5.95 |  | | 6.63 | | 6.22 | 7.06 |
| 64-68Y | 7.81 | 7.44 | 8.19 |  | 6.95 | 6.53 | 7.39 |  | 9.53 | 8.82 | 10.29 |  | | 7.54 | | 7.16 | 7.95 |  | | 6.58 | | 6.14 | 7.04 |  | | 9.30 | | 8.57 | 10.08 |
| ≥69Y | 10.22 | 9.73 | 10.73 |  | 9.04 | 8.52 | 9.60 |  | 12.67 | 11.65 | 13.77 |  | | 8.98 | | 8.51 | 9.47 |  | | 7.73 | | 7.23 | 8.26 |  | | 11.41 | | 10.43 | 12.48 |
| Stage 3 |  |  |  |  |  |  |  |  |  |  |  |  | |  | |  |  |  | |  | |  |  |  | |  | |  |  |
| 19-45Y | Ref. | Ref. | Ref. |  | Ref. | Ref. | Ref. |  | Ref. | Ref. | Ref. |  | | Ref. | | Ref. | Ref. |  | | Ref. | | Ref. | Ref. |  | | Ref. | | Ref. | Ref. |
| 45-48Y | 2.35 | 2.24 | 2.48 |  | 2.06 | 1.92 | 2.20 |  | 2.79 | 2.59 | 3.01 |  | | 2.54 | | 2.41 | 2.66 |  | | 2.27 | | 2.13 | 2.43 |  | | 2.90 | | 2.70 | 3.12 |
| 49-53Y | 3.75 | 3.59 | 3.92 |  | 2.86 | 2.69 | 3.04 |  | 5.07 | 4.75 | 5.42 |  | | 4.20 | | 4.01 | 4.39 |  | | 3.32 | | 3.13 | 3.53 |  | | 5.42 | | 5.07 | 5.79 |
| 54-58Y | 6.85 | 6.56 | 7.16 |  | 5.61 | 5.29 | 5.95 |  | 8.67 | 8.12 | 9.26 |  | | 8.05 | | 7.70 | 8.41 |  | | 6.86 | | 6.46 | 7.28 |  | | 9.61 | | 9.00 | 10.27 |
| 59-63Y | 11.48 | 10.97 | 12.01 |  | 10.17 | 9.57 | 10.80 |  | 13.43 | 12.54 | 14.40 |  | | 13.77 | | 13.14 | 14.43 |  | | 12.74 | | 11.96 | 13.57 |  | | 15.25 | | 14.21 | 16.38 |
| 64-68Y | 17.47 | 16.58 | 18.41 |  | 15.98 | 14.91 | 17.13 |  | 19.51 | 18.00 | 21.15 |  | | 21.79 | | 20.60 | 23.05 |  | | 20.00 | | 18.56 | 21.55 |  | | 23.97 | | 22.00 | 26.11 |
| ≥69Y | 28.54 | 27.09 | 30.06 |  | 24.65 | 23.07 | 26.34 |  | 34.90 | 32.07 | 37.97 |  | | 34.34 | | 32.46 | 36.32 |  | | 29.31 | | 27.29 | 31.48 |  | | 42.70 | | 38.99 | 46.77 |
| Stage 4 |  |  |  |  |  |  |  |  |  |  |  |  | |  | |  |  |  | |  | |  |  |  | |  | |  |  |
| 19-45Y | Ref. | Ref. | Ref. |  | Ref. | Ref. | Ref. |  | Ref. | Ref. | Ref. |  | | Ref. | | Ref. | Ref. |  | | Ref. | | Ref. | Ref. |  | | Ref. | | Ref. | Ref. |
| 45-48Y | 2.36 | 2.19 | 2.55 |  | 1.81 | 1.62 | 2.03 |  | 3.02 | 2.72 | 3.34 |  | | 2.58 | | 2.40 | 2.78 |  | | 2.06 | | 1.85 | 2.30 |  | | 3.20 | | 2.90 | 3.53 |
| 49-53Y | 3.98 | 3.73 | 4.25 |  | 2.85 | 2.58 | 3.14 |  | 5.40 | 4.94 | 5.91 |  | | 4.54 | | 4.26 | 4.84 |  | | 3.34 | | 3.04 | 3.67 |  | | 6.01 | | 5.50 | 6.56 |
| 54-58Y | 7.22 | 6.79 | 7.69 |  | 4.94 | 4.51 | 5.42 |  | 10.11 | 9.27 | 11.02 |  | | 8.50 | | 8.00 | 9.04 |  | | 5.93 | | 5.41 | 6.49 |  | | 11.66 | | 10.70 | 12.70 |
| 59-63Y | 12.63 | 11.86 | 13.45 |  | 11.69 | 10.73 | 12.75 |  | 13.67 | 12.47 | 14.98 |  | | 15.19 | | 14.26 | 16.17 |  | | 14.10 | | 12.93 | 15.39 |  | | 16.41 | | 14.98 | 17.97 |
| 64-68Y | 17.09 | 15.93 | 18.34 |  | 13.69 | 12.40 | 15.11 |  | 21.45 | 19.38 | 23.74 |  | | 21.92 | | 20.40 | 23.56 |  | | 17.73 | | 16.03 | 19.61 |  | | 27.28 | | 24.59 | 30.26 |
| ≥69Y | 31.25 | 29.22 | 33.42 |  | 24.83 | 22.70 | 27.16 |  | 40.59 | 36.65 | 44.94 |  | | 38.57 | | 35.99 | 41.33 |  | | 30.19 | | 27.54 | 33.10 |  | | 51.40 | | 46.23 | 57.15 |
| Stage 5 |  |  |  |  |  |  |  |  |  |  |  |  | |  | |  |  |  | |  | |  |  |  | |  | |  |  |
| 19-45Y | Ref. | Ref. | Ref. |  | Ref. | Ref. | Ref. |  | Ref. | Ref. | Ref. |  | | Ref. | | Ref. | Ref. |  | | Ref. | | Ref. | Ref. |  | | Ref. | | Ref. | Ref. |
| 45-48Y | 2.04 | 1.88 | 2.22 |  | 1.41 | 1.25 | 1.60 |  | 2.99 | 2.67 | 3.36 |  | | 2.20 | | 2.03 | 2.38 |  | | 1.53 | | 1.36 | 1.73 |  | | 3.18 | | 2.84 | 3.55 |
| 49-53Y | 3.42 | 3.19 | 3.67 |  | 2.21 | 1.99 | 2.46 |  | 5.32 | 4.80 | 5.88 |  | | 3.88 | | 3.62 | 4.16 |  | | 2.51 | | 2.27 | 2.78 |  | | 5.92 | | 5.37 | 6.54 |
| 54-58Y | 6.51 | 6.08 | 6.96 |  | 3.92 | 3.55 | 4.32 |  | 10.55 | 9.58 | 11.61 |  | | 7.58 | | 7.09 | 8.10 |  | | 4.62 | | 4.19 | 5.09 |  | | 12.01 | | 10.93 | 13.20 |
| 59-63Y | 9.12 | 8.50 | 9.78 |  | 7.18 | 6.53 | 7.90 |  | 11.98 | 10.79 | 13.29 |  | | 11.17 | | 10.42 | 11.98 |  | | 9.13 | | 8.30 | 10.05 |  | | 14.10 | | 12.72 | 15.63 |
| 64-68Y | 14.15 | 13.11 | 15.28 |  | 10.54 | 9.49 | 11.71 |  | 19.81 | 17.70 | 22.19 |  | | 17.92 | | 16.58 | 19.38 |  | | 13.28 | | 11.93 | 14.79 |  | | 25.11 | | 22.40 | 28.15 |
| ≥69Y | 21.13 | 19.62 | 22.75 |  | 14.85 | 13.46 | 16.37 |  | 32.14 | 28.67 | 36.03 |  | | 26.01 | | 24.11 | 28.06 |  | | 18.13 | | 16.40 | 20.03 |  | | 40.12 | | 35.68 | 45.11 |

Note: Stage -1 and 19-45y considered as reference group; values were adjusted for sex and years; sex was removed in sex specific analyses; OR: Odds ratio; CI: Confidence interval; LL: Lower limit; UL: Upper limit; MDRD: Modification of diet in renal disease Y: Years
