# Supplementary material for: Renal insufficiency among urban populations in Bangladesh: A decade of laboratory-based observations
Source: PLoS One. 2019 Apr 4;14(4):e0214568. doi: 10.1371/journal.pone.0214568 (PMC6448896; doi:10.1371/journal.pone.0214568)
Supplement: S8 Table — (DOCX) [file pone.0214568.s008.docx]

**S8 Table:** Overall and sex-specific blood glucose adjusted risk of renal insufficiency following CKD-EPI equation

|  | Overall (N=30582) | | |  | Male (N=123957) | | |  | Female (N=94931) | | |
| --- | --- | --- | --- | --- | --- | --- | --- | --- | --- | --- | --- |
|  | OR | 95% CI | |  | OR | 95% CI | |  | RR | [95% Conf. | |
| Stage-2 |  | LL | UL |  |  | LL | UL |  |  | LL | UL |
| 19-45Y | Ref. | Ref. | Ref. |  | Ref. | Ref. | Ref. |  | Ref. | Ref. | Ref. |
| 45-48Y | 2.49 | 2.24 | 2.76 |  | 2.38 | 2.10 | 2.69 |  | 2.89 | 2.35 | 3.56 |
| 49-53Y | 3.74 | 3.39 | 4.13 |  | 3.46 | 3.08 | 3.89 |  | 4.70 | 3.89 | 5.68 |
| 54-58Y | 6.90 | 6.26 | 7.60 |  | 6.54 | 5.82 | 7.35 |  | 8.40 | 6.98 | 10.11 |
| 59-63Y | 10.05 | 9.07 | 11.14 |  | 9.68 | 8.54 | 10.98 |  | 12.28 | 10.14 | 14.88 |
| 64-68Y | 17.59 | 15.52 | 19.93 |  | 18.60 | 15.81 | 21.89 |  | 20.68 | 16.70 | 25.61 |
| ≥69Y | 70.33 | 58.85 | 84.06 |  | 80.25 | 63.01 | 102.22 |  | 75.18 | 56.70 | 99.69 |
| Stage-3 |  |  |  |  |  |  |  |  |  |  |  |
| 19-45Y | Ref. | Ref. | Ref. |  | Ref. | Ref. | Ref. |  | Ref. | Ref. | Ref. |
| 45-48Y | 2.62 | 2.13 | 3.21 |  | 2.43 | 1.80 | 3.30 |  | 2.74 | 2.07 | 3.63 |
| 49-53Y | 5.44 | 4.55 | 6.50 |  | 5.05 | 3.89 | 6.56 |  | 5.68 | 4.45 | 7.26 |
| 54-58Y | 13.27 | 11.23 | 15.69 |  | 15.37 | 12.11 | 19.51 |  | 11.42 | 9.03 | 14.44 |
| 59-63Y | 21.83 | 18.44 | 25.84 |  | 26.79 | 21.08 | 34.05 |  | 17.72 | 13.96 | 22.51 |
| 64-68Y | 53.92 | 45.05 | 64.54 |  | 84.66 | 65.54 | 109.36 |  | 32.91 | 25.49 | 42.48 |
| ≥69Y | 241.25 | 193.78 | 300.35 |  | 371.74 | 272.43 | 507.26 |  | 148.93 | 109.10 | 203.30 |
| Stage-4 |  |  |  |  |  |  |  |  |  |  |  |
| 19-45Y | Ref. | Ref. | Ref. |  | Ref. | Ref. | Ref. |  | Ref. | Ref. | Ref. |
| 45-48Y | 3.47 | 2.36 | 5.08 |  | 4.57 | 2.39 | 8.74 |  | 2.95 | 1.83 | 4.76 |
| 49-53Y | 6.14 | 4.35 | 8.66 |  | 8.30 | 4.59 | 15.03 |  | 5.18 | 3.38 | 7.93 |
| 54-58Y | 14.01 | 10.14 | 19.37 |  | 15.04 | 8.44 | 26.80 |  | 13.36 | 9.02 | 19.77 |
| 59-63Y | 24.47 | 17.75 | 33.73 |  | 37.07 | 21.27 | 64.59 |  | 19.08 | 12.83 | 28.37 |
| 64-68Y | 53.04 | 38.15 | 73.74 |  | 100.50 | 57.20 | 176.58 |  | 33.73 | 22.35 | 50.90 |
| ≥69Y | 272.60 | 193.12 | 384.78 |  | 533.60 | 299.44 | 950.86 |  | 159.93 | 102.96 | 248.40 |
| Stage-5 |  |  |  |  |  |  |  |  |  |  |  |
| 19-45Y | Ref. | Ref. | Ref. |  | Ref. | Ref. | Ref. |  | Ref. | Ref. | Ref. |
| 45-48Y | 1.97 | 1.25 | 3.10 |  | 1.89 | 0.91 | 3.90 |  | 2.02 | 1.13 | 3.61 |
| 49-53Y | 3.21 | 2.15 | 4.79 |  | 3.49 | 1.87 | 6.53 |  | 3.06 | 1.82 | 5.15 |
| 54-58Y | 9.82 | 6.95 | 13.87 |  | 8.35 | 4.74 | 14.70 |  | 10.72 | 6.91 | 16.64 |
| 59-63Y | 16.41 | 11.64 | 23.14 |  | 15.27 | 8.77 | 26.58 |  | 17.02 | 10.97 | 26.41 |
| 64-68Y | 33.10 | 23.15 | 47.33 |  | 42.64 | 24.29 | 74.87 |  | 27.02 | 17.01 | 42.90 |
| ≥69Y | 128.90 | 88.64 | 187.44 |  | 172.53 | 97.06 | 306.69 |  | 97.57 | 59.50 | 160.01 |

CKD-EPI: Chronic Kidney Disease Epidemiology Collaboration; CI: Confidence interval; LL: Lower limit, OR: odds ratio; UL: Upper limit; Y: Years
